# LRRCE: a leucine-rich repeat cysteine capping motif unique to the chordate lineage

**DOI:** 10.1186/1471-2164-9-599

**Published:** 2008-12-12

**Authors:** Hosil Park, Julie Huxley-Jones, Ray P Boot-Handford, Paul N Bishop, Teresa K Attwood, Jordi Bella

**Affiliations:** 1 Wellcome Trust Centre for Cell-Matrix Research, Faculty of Life Sciences, University of Manchester, Manchester, M13 9PT, UK; 2 Faculty of Life Sciences and School of Computer Science, University of Manchester, Manchester, M13 9PT, UK; 3 Computational Biology, Molecular Discovery Research, GlaxoSmithKline Pharmaceuticals, Harlow, Essex, CM19 5AW, UK

## Abstract

**Background:**

The small leucine-rich repeat proteins and proteoglycans (SLRPs) form an important family of regulatory molecules that participate in many essential functions. They typically control the correct assembly of collagen fibrils, regulate mineral deposition in bone, and modulate the activity of potent cellular growth factors through many signalling cascades. SLRPs belong to the group of extracellular leucine-rich repeat proteins that are flanked at both ends by disulphide-bonded caps that protect the hydrophobic core of the terminal repeats. A capping motif specific to SLRPs has been recently described in the crystal structures of the core proteins of decorin and biglycan. This motif, designated as LRRCE, differs in both sequence and structure from other, more widespread leucine-rich capping motifs. To investigate if the LRRCE motif is a common structural feature found in other leucine-rich repeat proteins, we have defined characteristic sequence patterns and used them in genome-wide searches.

**Results:**

The LRRCE motif is a structural element exclusive to the main group of SLRPs. It appears to have evolved during early chordate evolution and is not found in protein sequences from non-chordate genomes. Our search has expanded the family of SLRPs to include new predicted protein sequences, mainly in fishes but with intriguing putative orthologs in mammals. The chromosomal locations of the newly predicted SLRP genes would support the large-scale genome or gene duplications that are thought to have occurred during vertebrate evolution. From this expanded list we describe a new class of SLRP sequences that could be representative of an ancestral SLRP gene.

**Conclusion:**

Given its exclusivity the LRRCE motif is a useful annotation tool for the identification and classification of new SLRP sequences in genome databases. The expanded list of members of the SLRP family offers interesting insights into early vertebrate evolution and suggests an early chordate evolutionary origin for the LRRCE capping motif.

## Background

The leucine-rich repeat (LRR) is a widespread structural motif of 20–30 amino acids easily identifiable at the primary structure level by the characteristic 11-residue hallmark sequence L*xx*L*x*L*xx*N*x*L, where *x *means any amino acid and the consensus Leu and Asn positions are often substituted by other hydrophobic residues such as Ile, Val, Phe, Cys, etc [1-5]. Proteins with LRR-architecture typically contain two or more LRRs in tandem and have been identified in all life forms, from viruses to eukaryotes [6]. The continuously expanding LRR superfamily includes intracellular, extracellular and membrane-attached proteins characterized by a common modular architecture specially suited to favour protein-protein interactions [1-3,5,7-9]. These proteins participate in a variety of important biological functions, including among others cell adhesion and signalling, platelet aggregation, neural development, extracellular matrix assembly, bacterial pathogenicity, disease resistance and immune response [10-20]. LRR-containing proteins and domains form curved solenoid structures where each repeat is a turn of the solenoid. The concave side of the solenoid is defined by a parallel β-sheet interwoven with a variety of structures in the convex side which include α helices, 3_10 _helices, polyproline II helices, tandems of β turns and short β strands [1-5,9,21]. The biological roles of LRR proteins and domains typically relate to their ability to engage in protein-protein interactions. However, some family members recognize other ligand types such as nucleic acids, lipopolysaccharides, lipopeptides, and even small compounds such as auxins [19,22-27]. The sites for ligand recognition map preferentially but not exclusively to the concave sites of the LRR arched structures, as demonstrated by several crystal structures of LRR proteins in complex with their ligands (see [5] for a recent review). Recently, some LRR proteins have been shown to form highly stable dimers through their concave side [28-31] raising the possibility of alternative scenarios where LRR dimers are either the functional units or latent forms that require dissociation prior to ligand binding [32].

A distinct group of LRR proteins from the extracellular matrix forms the family known as small leucine-rich repeat proteins and proteoglycans (SLRPs) [10,32-34]. These molecules are emerging as an important family or regulatory proteins with still undiscovered functions. They typically control the correct assembly of collagen fibrils, regulate mineral deposition in bone, and modulate the activity of potent cellular growth factors through signal transduction [10,33-35]. SLRPs have in common clusters of cysteine residues flanking their LRR domains at both N- and C-termini. The crystal structures of the two most studied SLRPs, decorin and biglycan, have been recently determined [29,31].

SLRPs have been traditionally classified into three classes (I, II and III) depending on their gene organisation, number of LRRs and spacing of cysteine residues at the amino-terminal cluster [10,32,33]. Other LRR molecules have been subsequently added to the family and two additional, non-canonical classes IV and V have been defined [34]. Class IV and V SLRPs show clear differences with those of the three first classes in number of repeats and internal repeat structure [32,34,36-39]; their classification as SLRPs is due to functional similarity with canonical SLRPs, extracellular location, and presence of cysteine clusters flanking the LRR domain.

**Figure 1:**
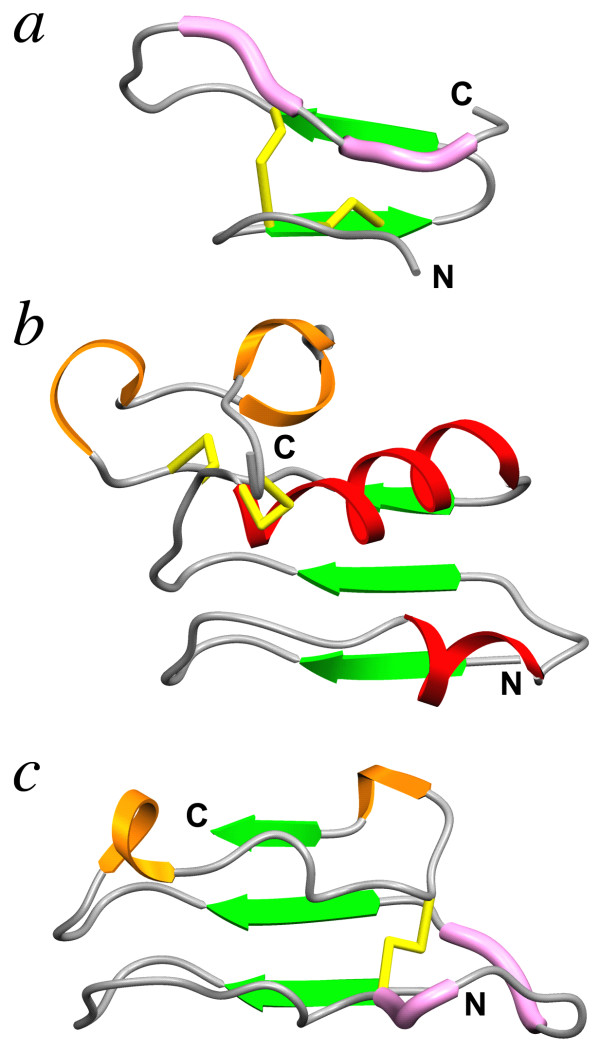
**Ribbon diagrams of different cysteine-capping motifs in LRR structures, viewed from the convex side of the LRR domains: (*a*) the LRRNT capping motif in the crystal structure of bovine decorin **[29]**, PDB code **1XKU**; (*b*) the LRRCT capping motif in the crystal structure of the Nogo receptor ectodomain **[46]**, PDB code **1OZN**; (*c*) the LRRCE capping motif in the crystal structure of bovine decorin **[29]. The different secondary structure elements are identified as follows: green arrows, β-strands; red ribbons, α-helices; orange ribbons, 3_10 _helices and β-turns; pink tubes, short polyproline II segments; yellow sticks, disulphide bonds. The N- and C-terminal ends in each panel are indicated. (Reproduced from [5] with permission from Birkhäuser Verlag AG)

Many LRR proteins other than SLRPs are flanked at the N- and C-termini by disulphide-bonded caps that are thought to protect the hydrophobic core of the first and last LRRs [2,3]. Both N-terminal and C-terminal capping motifs are described in databases for protein domain identification and analysis such as SMART [40], Pfam [41] and InterPro [42] (Table 1). In the LRR N-terminal capping motif (LRRNT), a single β-strand runs antiparallel to the main β-sheet and is followed by a short LRR of 20 or 21 residues (Figure 1a). The consensus sequence contains 4 cysteines in a C*x*_*n*_C*x*C*x*_*m*_C pattern, with *n *and *m *being variable numbers.

**Table 1:** LRR cysteine-capping motifs described in the protein domain databases InterPro (16.2) [42], SMART (5.1) [40] and Pfam (22.0) [41], with current numbers of matches.

Name	Description	Database	Accession	Matches
**LRRNT**	N-terminal capping	SMART	SM00013	5596
		InterPro	IPR000372	2610
		Pfam	PF01462	1572

**LRRNP**	N-terminal capping (plant specific)	InterPro	IPR013210	2517
		Pfam	PF08263	1326

**LRRCT**	C-terminal capping	SMART	SM00082	4715
		InterPro	IPR000483	1476
		Pfam	PF01463	399

The cysteines form a disulphide knot that connects the antiparallel β strand to the first LRR. This motif is characteristic of all proteins from the SLRP family as well as secreted or membrane-bound LRR proteins. Its main structural elements appear to be maintained irrespective of the number and spacing of cysteines [32]. A variation of the LRRNT capping motif specific to plants has also been described (LRRNP, Table 1). Its architecture and disulphide-bonding topology differs from that of LRRNT, as observed in the crystal structure of a polygalacturonase inhibitor from *Phaseolus vulgaris *[43].

The LRR C-terminal capping motif (LRRCT) contains normally four cysteines that stabilize the local structure with two disulphide bonds [44-47]. Characteristic of this motif is an α helix that covers the hydrophobic core of the last LRR (Figure 1b). This capping motif seems to occur slightly less often than LRRNT (Table 1) and appears to be exclusive to animal proteins. Many cysteine-capped LRR proteins have been automatically annotated as having either N-terminal or C-terminal capping structures, although close inspection of their sequences shows that in many cases both capping motifs are actually present.

In early sequence analyses, Kajava and Kobe classified the disulphide-bonded C-terminal capping motifs in four different subfamilies, named CF1 to CF4 [2,3]. Sequences from the CF1 subfamily contain four cysteine residues and are the ones typically detected by current LRRCT database descriptors in automatic sequence annotation (Table 1). The CF2 subfamily is characterized by only two cysteines and was defined for some members of the SLRP family. The CF3 and CF4 subfamilies are specific to G-protein coupled receptors and plants, respectively. Protein sequence databases do not include separate descriptors for these four subfamilies, and sequences from the CF2 subfamily are not recognized by current LRRCT descriptors. The structure of the C-terminal capping motif from the CF2 subfamily was elucidated in the crystal structures of the protein cores of decorin and biglycan [29,31], both being representative members of the SLRP family. This capping motif is structurally quite different from LRRCT [32]. It extends to the last two LRRs, which are connected by a single disulphide bond (Figure 1c). The second-to-last LRR appears to be longer than all the other ones, and in the crystal structures of decorin and biglycan is extended laterally from the main LRR fold [29,31]. We have previously named this longer, extended repeat as the "ear" repeat, and we will use the term LRRCE throughout this paper to designate the ear-containing LRR C-terminal capping motif. All extracellular LRR proteins currently classified as members of the SLRP family have N-terminal capping motifs of the LRRNT type, but their C-terminal motifs show variability. While chondroadherin and nyctalopin C-terminal sequences correspond to that of a typical LRRCT motif, the SLRPs from the canonical group (which here refers to classes I, II and III plus extracellular matrix protein 2, ECM2) show the LRRCE capping motif [29,32]. Podocan, a recent addition to the SLRP family and class V representative, does not have any C-terminal capping [32,36].

To investigate if the LRRCE motif is a common structural feature in other LRR proteins, we have defined characteristic sequence patterns based on a set of sequences from class I, II and III SLRPs and we have used those patterns in genome-wide searches. We present in this paper the results of this analysis.

## Results and discussion

### A comprehensive list of LRRCE-containing sequences

**Figure 2:**
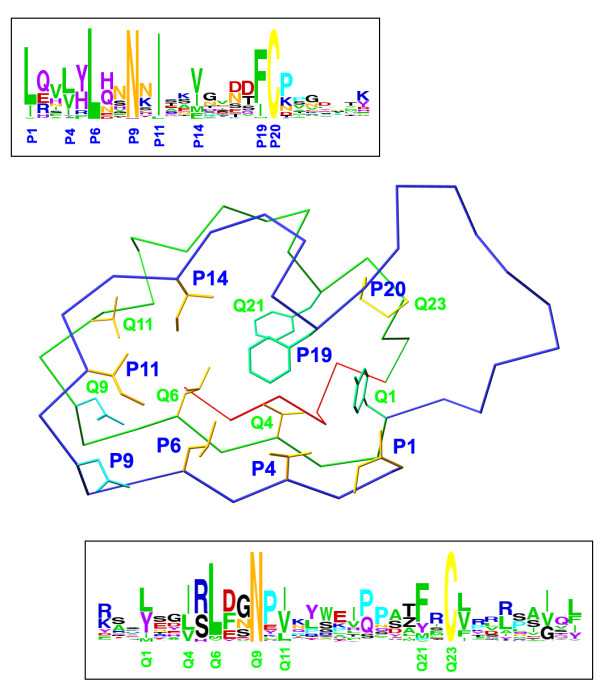
**Mapping of the regular expression pattern of the LRRCE motif on a skeletal representation of the LRRCE structure from bovine decorin **[29]. The motif includes the laterally extended ear repeat, shown as a Cα trace in blue, the following LRR (in green Cα trace), and the final β-strand closing the domain (red Cα trace). The regular expression pattern used in this study, written in *PROSITE *syntax [79], was: [LIV]-X(2)-[LVIYFMA]-X-[LIFM]-X(2)-[NH]-X-[ILVF]-X(2)-[VIMFLY]-X(4)-[FIMLV]-C-X(7,20)-[LYIMV]-X(2)-[ILVTMF]-X-[LVMI]-X(2)-N-X-[IVLMAFT]-X(8,9)-[FYMPVAIS]-X-C. In *PROSITE *syntax each conserved position is shown either as a single amino acid (e.g. C, N) or all possible amino acids for that position enclosed within brackets (e.g. [ILVF] indicates that such position is occupied by Ile, Leu, Val or Phe); each variable position is shown with a letter X. Numbers in parentheses indicate stretches of variable positions (e.g. X(7,20) indicates a stretch of between 7 and 20 variable amino acids). Amino acid preferences for each position are shown in two boxes in "weblogo" form [87]. The conserved sequence positions for the ear repeat on the LRRCE motif are designated as P1, P4, P6,..., P20, and those for the following LRR as Q1, Q4, Q6,..., Q23. The side chains show the amino acids occurring at these conserved positons in the structure of bovine decorin.

**Figure 3:**
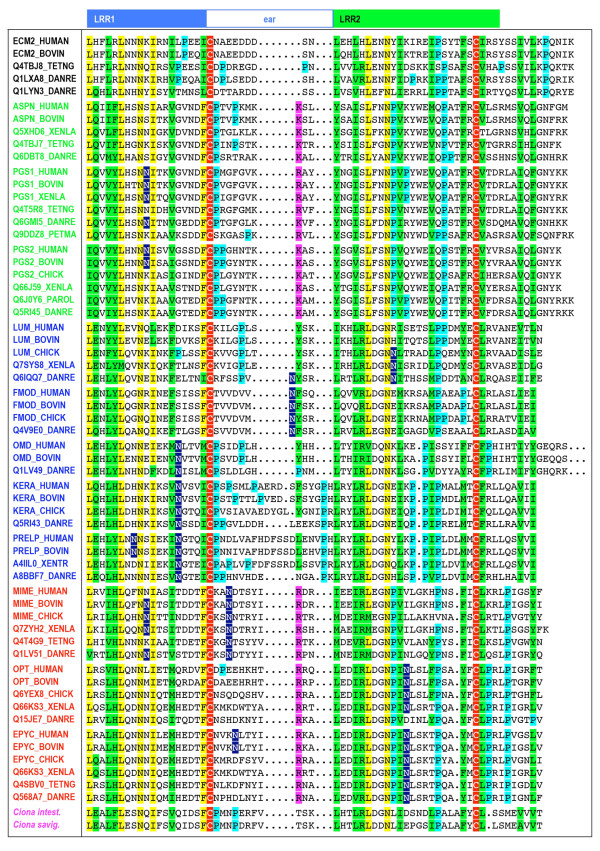
**Multiple sequence alignment of LRRCE motifs from a selected set of SLRP sequences from the UniProt non-redundant set**. Names for the sequences are those of their corresponding Swiss-Prot or TrEMBL entries. Members of the different classes are shown with their names in green (class I), blue (class II), red (class III) or black (ECM2 and similar proteins). Two sequences from early SLRPs in urochordates (*Ciona intestinalis *and *Ciona savigny*) are also included with their name in magenta (see text). The boxes on the top indicate the two consecutive repeats LRR1 and LRR2 that contain the LRRCE motif. The ear itself is included in the first repeat. Residue conservation colour scheme: conserved cysteines in red; conserved residues in yellow; partially conserved residues in green; conserved prolines in cyan; polar residues in conserved hydrophobic sites in magenta; potential sites of N-linked glycosylation in blue.

The final regular expression pattern for the LRRCE capping motif and its mapping to the three-dimensional structure of bovine decorin are shown in Figure 2. Using the LRRCE regular expression pattern, a total of 175 sequences were retrieved by ScanProsite from the UniProt database (Swiss-Prot release 55.1, TrEMBL release 38.1) [48]. Splice variants were excluded from the search and sequence duplicates were filtered, resulting in a non-redundant set of 110 UniProt sequences. Figure 3 shows a selection of aligned LRRCE sequences from this non-redundant set (an extended list of sequences including accession codes is available in Additional File 1), plus two sequences from urochordates (discussed later). The LRRCE regular expression pattern was accurate: all sequences in this UniProt set were of proteins with LRR architecture and had a repeat structure that identified them as canonical SLRPs, with the LRRNT capping motif at the N-terminus and the LRRCE capping motif at the C-terminus. The pattern was also comprehensive: all proteins known to belong to the canonical group of SLRPs or annotated as similar to them were retrieved in the search.

Sequences of LRRCE motifs from the UniProt non-redundant set were used in similarity searches with BLAST to retrieve additional LRRCE-containing sequences (see Methods). For most SLRPs, the LRRCE motifs are largely coded by the last exon from their corresponding genes. Thus, probe sequences just encompassing different LRRCE motifs were useful for quickly locating SLRP genes in genomes at early stages of annotation or for searching new SLRPs on the genomes of invertebrates and early chordates (see below). The extended list of 280 hits with their LRRCE sequences and accession codes, including those obtained from the NCBI and ENSEMBL databases, is provided in Additional File 1. The LRRCE regular expression pattern discussed earlier is consistent with all but seven of the sequences in this extended list. Three of these exceptions are incomplete sequences due to missing genomic data, and two more are predicted sequences with only one change with respect to the LRRCE pattern. Probably the only significant exceptions were the LRRCE sequences for the chicken and lizard homologues of ECM2, which contain an additional insertion (see Additional File 1). Many of the sequences found in similarity searches are from predicted model assemblies from genomes in early stages of annotation, and therefore some of the assignments should be considered preliminary.

### Structure of the LRRCE capping motif

The LRRCE motif encompasses the ear repeat, which is extended laterally, the LRR following it, and the final β-strand closing the domain (Figure 2). The regular expression pattern runs from the beginning of the ear repeat to the second cysteine residue. Sequence conservation in LRRCE motifs across the different SLRPs follows largely structural dictates, with the highly conserved positions mainly corresponding to the core hydrophobic or asparagine residues characteristic of the LRR architecture, plus the two cysteine residues that are connected by a disulphide bond (Figure 2). Several additional positions show distinct preferences for polar or charged amino acids. The corresponding residues in the bovine decorin structure participate in a network of stabilizing charge-charge and hydrogen-bonding interactions between repeats. Thus, it is likely that similar interactions will be conserved in the other LRRCE-containing proteins to impart stability to the capping motif. Residue conservation in the ear itself is comparatively poor between closely related proteins (for example, between decorin and biglycan), but higher for the same protein across species (see examples in Figures 3 and Additional File 1). This pattern of conservation suggests that the ear extension contributes to the functional specialisation of the canonical SLRPs. For most sequences, the ear extension is 11–13 residues long (from the first cysteine to the first residue of the second LRR), and only keratocan and PRELP sequences show consistently long extensions. A buried lysine residue shown to stabilize the ear conformation in the crystal structure of decorin [29] is conserved as lysine or arginine in all class I and III sequences, whereas in class II sequences the same position is occupied by an aromatic or leucine residue. Fibromodulin, osteoglycin and epiphycan sequences show conserved *N*-linked glycosylation sites in their ear extensions. Additional potential *N*-linked glycosylation sites appear with varying degrees of conservation on different regions of the two repeats forming the LRRCE motif.

### LRRCE motifs are always C-terminal

The LRRCE motif is a genuine C-terminal motif, with no instance of an equivalent architecture in the middle of an LRR domain or protein. The C-terminus is typically 9–15 residues away from the conserved second cysteine. The exception is the group of OMD sequences, which contain an extended C-terminal tail of about 60 residues after the LRRCE motif. This tail contains a stretch of negatively charged residues that presumably shares some functional role with the negatively charged glycosaminoglycan chains attached to the N-termini of decorin or biglycan, or the polyanionic stretches seen in the N-terminal region of asporin or preceding the LRR domain in ECM2.

The LRRCE motif can be related structurally to internal, disulphide-bonded LRR pairs that occasionally occur in LRR proteins. These LRRs do not show the lateral extensions characteristic of the LRRCE motifs. These intradomain, disulphide-bonded LRR pairs are much more widely distributed than LRRCE, as they occur in different LRR protein families from bacteria to humans. One such linkage can be seen in the three-dimensional structure of the ectodomain of Toll-like receptor 3 [49,50]. This structure is formed by a tandem of 25 LRRs capped by LRRNT and LRRCT motifs, and contains one internal disulphide-bonded LRR pair.

### The LRRCT and LRRCE structural motifs appear to be unrelated

There is no obvious relation between the LRRCT and LRRCE motifs, at least from sequences available to date. A conserved feature in the LRRCT capping motif is the presence of two or more Trp residues that contribute to maintain the hydrophobic core at the C-terminal end. A common sequence at the beginning of LRRCT motifs is NPW*x*C*x*C*x*_3_W*x*_3_W, where the second and third Trp residues are located on the inner side of the α-helix characteristic of the LRRCT structure (Figure 1b). Conversely, Trp residues are not particularly conserved in the LRRCE sequences (Figure 3 and Additional File 1), and the only Trp residue in the structure of bovine decorin [29] is exposed to the solvent and does not participate in the hydrophobic core of the C-terminal end of the LRR structure.

### A new class of canonical SLRPs, with sequences similar to ECM2

**Figure 4:**
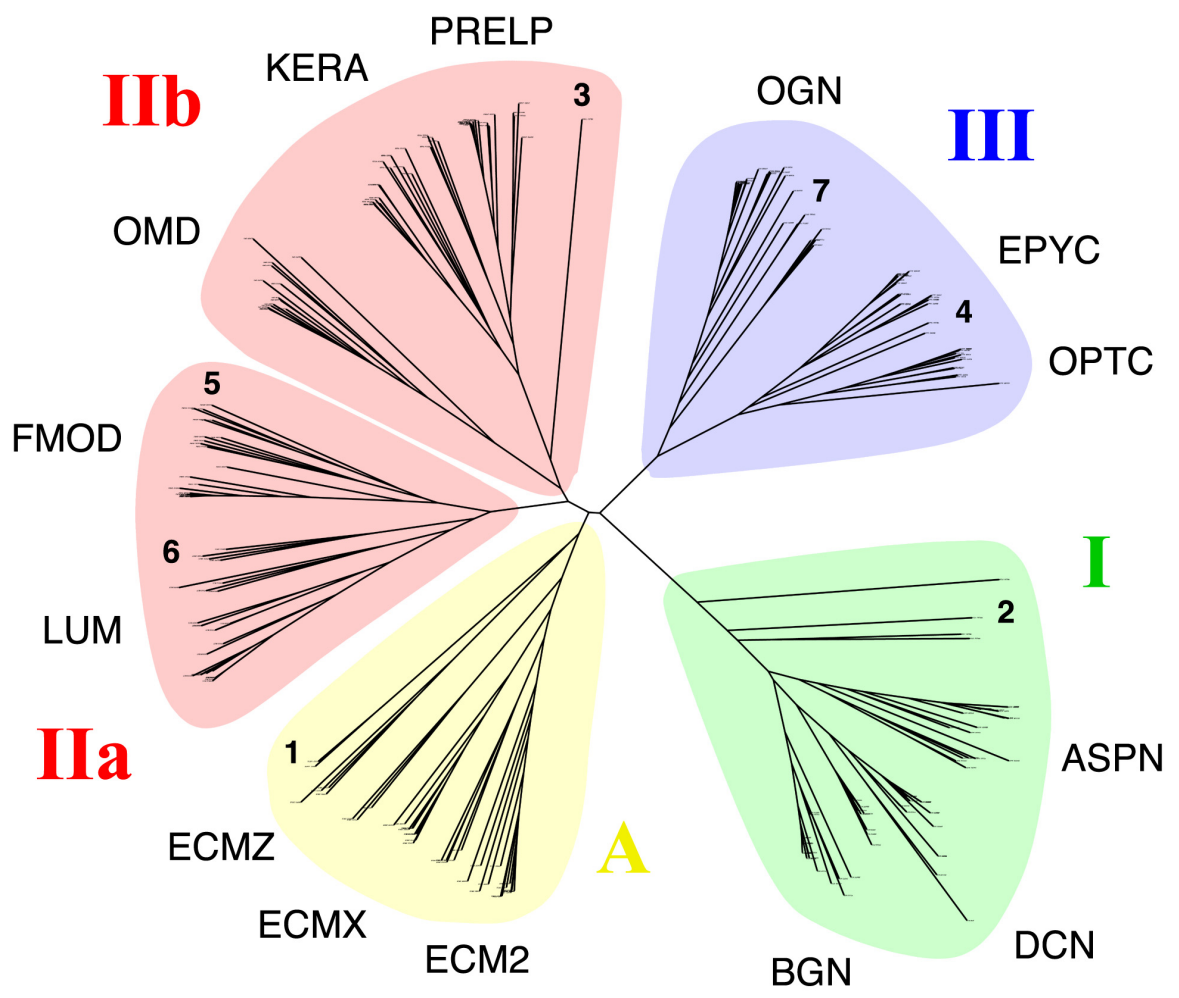
**Unrooted phylogenetic tree of an expanded set of LRRCE-containing sequences, including those from UniProt, NCBI and ENSEMBL databases**. Sequences group themselves in four main SLRP classes, and the class II branch has been split into two subclasses IIa and IIb. See text for the abbreviations describing each SLRP type. The positions of several sequences specifically discussed in the text are indicated with bold-type numerals: **1**, SLRP1 sequences from *Ciona intestinalis *and *Ciona savignyi*; **2**, biglycan-like (BGL) and decorin-like (DCL) sequences from sea lamprey *Petromyzon marinus*; **3**, keratocan-like (KERAL) sequence from lamprey; **4**, epiphycan-like (EPYL) sequence from lamprey; **5**, cluster of second copies of fibromodulin (FMOD2) exclusive to fish genomes; **6**, cluster of second copies of lumican (LUM2) exclusive to fish genomes; **7**, cluster of second copies of osteoglycin (OGN2) exclusive to fish genomes. This tree was calculated based on the sequence alignment of the LRRCE motifs. A larger version of this figure, with legible sequence names at the end of the phylogenetic tree branches, is provided as Additional File 2.

A cluster analysis of the extended set of LRRCE-containing sequences is shown in the form of a phylogenetic tree inferred from a multiple sequence alignment of the LRRCE motifs (Figure 4 and Additional File 2). The sequences group themselves in the canonical SLRP classes (I, II and III), plus an additional cluster containing ECM2 and related sequences. Class I includes asporin (ASPN), decorin (DCN) and biglycan (BGN) sequences. Class II contains two groups and could in fact be subdivided into IIa with lumican (LUM) and fibromodulin (FMOD) sequences, and IIb with osteomodulin (OMD), keratocan (KERA) and prolargin (PRELP) sequences. Class III includes sequences for opticin (OPTC), epiphycan (EPYC) and osteoglycin (OGN), also known as mimecan. The new "class A" is discussed later and includes the sequences of extracellular matrix protein 2 (ECM2), ECM2-like protein from the X chromosome (ECMX), ECM2-like predicted proteins upstream of the DCN gene in fish genomes (ECMZ), and the small leucine-rich repeat protein from *Ciona intestinalis *and *Ciona savignyi *(SLRP1) (see below). The same clustering was obtained using the complete SLRP sequences (not shown), indicating that the LRRCE sequences on their own are useful for canonical group SLRP classification.

### The LRRCE motif is unique to chordates

Given the exclusive vertebrate lineage of all sequences obtained in the UniProt set, searches with LRRCE sequences were carried out against several invertebrate genomes including *Drosophila melanogaster *(fruit fly), *Apis mellifera *(honeybee) [51], *Anopheles gambiae *(malaria mosquito), *Aedes aegypti *(yellowfever mosquito) [52], *Caenorhabditis elegans *(worm) and *Strongylocentrotus purpuratus *(sea urchin) [53]. Of particular interest was the genome of the sea urchin, as this organism appears to have retained some of the genes later observed only in vertebrates [53]. All the genomes investigated have large numbers of LRR proteins, many of those with LRRNT capping motifs. However, the searches failed to produce any true match of LRR proteins containing LRRCE motifs in these invertebrate genomes. Two LRR protein sequences from mosquito (Q16VM2_AEDAE and Q17NB1_AEDAE in TrEMBL) are currently annotated (incorrectly) as putative lumicans, although they lack recognizable LRRNT caps and neither their repeat lengths nor amino acid sequences correspond to those of lumican proteins. Nonchordate LRR protein sequences are often homologous to non-SLRP proteins such as toll-like receptors or slit proteins, and C-terminal disulphide-capping often occurs through the more common LRRCT type.

### LRRCE motifs in early chordates: implications for SLRP evolution

**Figure 5:**
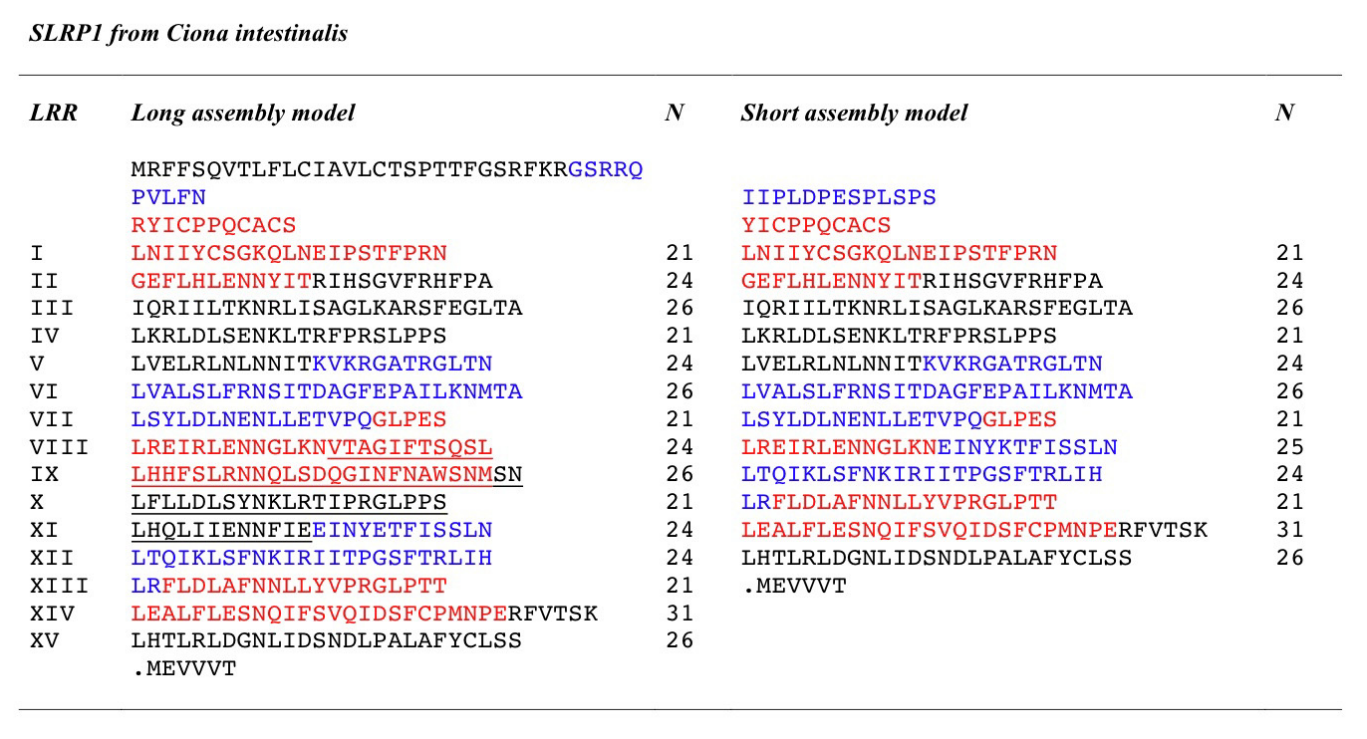
**Two different gene assembly models for the only LRRCE-containing sequence in *****Ciona intestinalis***. Sequences encoded by separate exons are shown in different colours (red-black-blue) for clarity. The long model assembly (left) contains 8 exons and 15 LRRs in its LRR domain. The same gene assembly model is used for the homologous protein in *Ciona savigny*. The short model assembly on the right contains 7 exons and 12 LRRs in its LRR domain; one and a half exons are skipped resulting in the removal of the underlined amino acids from the long form. Both models were generated using prediction algorithms. The short model was part of the first draft for the *Ciona intestinalis *genome [55] (JGI assembly version 1.0, ci148160), but was later withdrawn in JGI version 2.0 in favour of the longer model. Available EST data (see gene and transcript entries ENSCING00000012194, ENSCINT00000023142 in the ENSEMBL database), has confirmed the long assembly model with 15 LRRs.

Similarity searches using LRRCE sequences were then carried out against the genomes of the early chordates amphioxus (lancelet), *Branchiostoma floridae *[54], and the ascidians *Ciona intestinalis *[55] and *Ciona savignyi *[56]. Only one gene containing the LRRCE motif was found for each *Ciona *species. This gene, referred to here as SLRP1, has been proposed as representative of the ancestor of all modern canonical SLRPs [57]. Two model assemblies have been proposed for the *Ciona intestinalis *gene resulting in proteins with different numbers of LRRs (Figure 5). These two assemblies only differ in the prediction of one additional exon-intron boundary in the short model, which results in the skipping of one and a half exons from the long model. The long model assembly for the *Ciona intestinalis *SLRP1 gene has been confirmed by EST data (Figure 5). The resulting protein sequence has 15 LRRs in which repeats alternate their lenghts following a predominant 21-24-26 pattern; this repeat structure is very similar to that seen in the ECM2 sequences from vertebrates [32]. The cluster analysis of LRRCE motifs (Figure 4) places the SLRP1 sequences of *Ciona intestinalis *and *Ciona savigny *in the same group as ECM2 and related sequences. The alternating pattern of repeat lengths (21-24-26) is common to SLRP1 as well as a *Ciona *gene representative of the podocan ancestor [57], suggesting that such an alternating repeat sequence was already present in a common precursor of these two lineages. This observation is consistent with the concept of tandem LRR supermotifs characteristic of the evolutionary history of the SLRP family [58].

An interesting possibility is that shorter, 12-LRR SLRPs (such as these from classes I and II), originated by exon skipping from an ancestral SLRP gene with 15 LRRs similar to SLRP1 from *Ciona*, in a manner illustrated by the short model assembly shown in Figure 5. Such exon skipping would have occurred after duplication of this ancestral SLRP gene. The two genes could have later evolved after additional tandem and large-scale duplication events into two independent lineages, one for ECM2 and ECM2-like proteins (discussed below), and another for the class I and class II SLRPs. Class III SLRPs would have originated by further exon skipping on the class I and II ancestor, after its divergence from the ECM2 lineage ancestor.

No gene containing an LRRCE motif could be detected in the currently available release of the amphioxus genome (*Branchiostoma floridae*, JGI version 1.0) [54]. Sequence similarity searches using the protein sequences of the three SLRP proteins from *Ciona *(Figure 5 and [57]) produced putative orthologues of podocan and chondroadherin (data not shown), and partial hits to many non-SLRP protein sequences with LRR architecture (several cell-surface receptors, slit-like proteins, etc). However, the searches failed to return any clear orthologues of the canonical SLRPs or any sequence containing a LRRCE motif. Queries using sequences conforming to LRRCE motifs from *Ciona *yielded no hits either. The apparent absence of LRRCE-containing genes in *Brachiostoma *is intriguing. Cephalochordates (amphioxus) have been traditionally considered to be the primitive chordates that most resemble vertebrates, but this view has been very recently contradicted by the genome sequence data that suggests that tunicates (which include the ascidians), are more closely related to vertebrates than cephalochordates [59]. This finding raises the possibility that the first LRRCE-containing SLRP genes appeared after the divergence of cephalochordates from the rest of the tunicate-vertebrate lineage. Alternatively, the gene equivalent to SLRP1 from *Ciona *was already present in a common ancestor of all chordates but may have been lost in the cephalochordate lineage.

### Agnathans show already an expanded set of SLRP sequences

Searches for LRRCE motifs against the current release of the sea lamprey genome *Petromyzon marinus *(Genome Sequence Center, Washington University) produced six LRRCE-containing sequences. Two of them correspond to previously reported biglycan-like proteins [60], whereas the four additional hits correspond to predicted partial sequences similar to decorin (two sequences), epiphycan (one sequence) and keratocan (one sequence). We have named these sequences biglycan-like proteins 1 and 2 (BGL1 and BGL2), decorin-like proteins 1 and 2 (DCL1 and DCL2), epiphycan-like protein (EPYL) and keratocan-like protein (KERAL) (Table 2). Although these data should still be considered preliminary, the sequences represent the earliest examples to date of class I, II and III SLRPs, suggesting that the divergence of the SLRP ancestor into three classes, following gene duplication, occurred before the lamprey-gnathostome split. Completion of the lamprey genome and possible identification of additional copies of EPYL and KERAL genes will clarify the relation between individual SLRP gene duplications and the large-scale gene or genome duplication that is thought to have occurred before the divergence of lampreys from gnathostomes [57,61,62].

**Table 2:** Representative examples of class I, II and III SLRP sequences in sea lamprey.

**LRR**	**BGL2_PETMA (class I)**	***N***	**KERAL_PETMA (class II)**	***N***
	...PDASCPFGCQCS		...PPLCPVACYCPPDH	
I	ARVVQCSDLGLVSVPQAIPKD	21	PGAIYCDGRELHDVPRIPAR	20
II	ARLLDLQNNKITEIKQDDFKGLNK	24	VRFAYFQNNNIEALSECDLRDAGG	24
III	LYALYLVNNLISKVHPKAFAPLSS	24	LLGLNLDDNVLTSPTLSQDTLRSLRH	26
IV	LDKLYISHNQLTEVPGSMPSS	21	LSQLHLQRNQLTEVPLGLPAS	21
V	LVELRIHENNIKKIPKDAFSGMKR	24	LEDLRLGQNRIALVPKGAFARLSR	24
VI	LHALEMGGNPLQSTGIEVGAFEGLER	26	LRMLDLSANRLQVLRDDAFAGLSA	24
VII	LVYVRVSDSKLARIPKDLPNS	21	LVQLNLAENRLRAMPPKPPSGL	22
VIII	IQELHLEHNQITALEQEDLIRYPL	24	LYQLILCDNVIESIPDNYLASFPR	24
IX	IHRLGLSYNQIKVIQNGSLETCPH	24	LAWLDLGKNALGTRREKRTGIPERAFISRA	30
X	LRELHLDSNVLTQVPPGLAFLKH	23	LLNLRLSANHLQHVPAFHGN	20
XI	LQVVYLHSNKIAAVKSDDFCSKGASPKRVL	30	LVQLHLDENDIEDVNTTALCRPEGRESSR	29
XII	YSGISLFDNPVNYWDVPPSAFRCVASR	27	LSYFRLDKNPIMESPQAPLMHCFPY	25
	.SAVQFSQNFRK		LQPMF	

**LRR**	**EPYL_PETMA (class III)**	***N***		

	...MPTCLLCSCV			
I	HGSVYCDDLELDSVPPLPKD	20		
II	TVYLYARFNKIRTLRKKDLSGYAQ	24		
III	LKRVDLSSNGLTSVEAGALAQLPA	24		
IV	LEEVLLAGNELVALPELPPA	20		
V	TRRLDARQNHVTSKGVAADMFEKMKQ	26		
VI	LEYLYLSDNQLDFIPVPLPDS	21		
VII	LRVLHLQNNNIQQIREDTFCKPKELSYFRKA	31		
VIII	LEDVRLDGNPVNLSDAPEAYTCLPR	25		
	IPTGATF			

Repeats are numbered in roman numerals, and *N *is the length of each repeat. Sequences amino terminal to the LRRNT cluster have not been included.

### The LRRCE motif is a useful annotation tool for extending the SLRP family

The LRRCE capping motif is useful in sequence annotation as it appears to be exclusive to the canonical group of SLRPs. The presence of an LRRCE motif in a newly predicted protein sequence is sufficient for its quick identification and classification as a member of the canonical group of SLRPs (and also into one of its classes). The amino acid sequences of LRRCE motifs can be used as probes in similarity searches against translated genomic databases (TBLASTN). These searches can identify the locations and partial sequences of new putative SLRP genes in genomes at different levels of completion (as shown with the lamprey example). Using these probes, it is also possible to detect exons or domains that are missing in current assembly models of SLRP genes but nevertheless present in the genome (data not shown).

**Figure 6:**
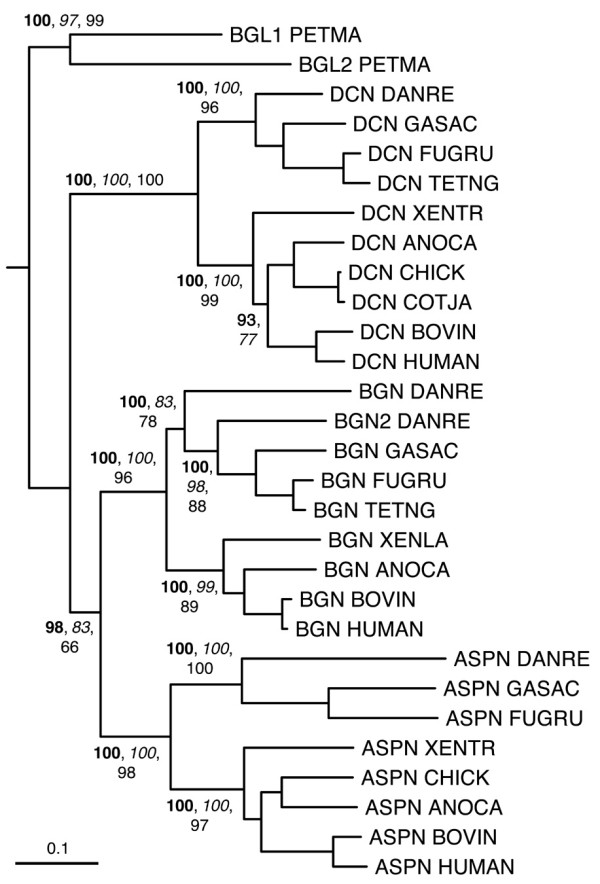
**Phylogenetic relationships of class I SLRPs, inferred from the multiple sequence alignment of LRR domains from a reduced set of SLRP sequences (see Methods)**. The tree has been rooted using the BGL lamprey sequences as outgroup. A second BGN sequence (BGN2) has been identified in the zebrafish genome but not yet in other fishes. Clade proability values higher than 60% are indicated, bayesian estimates in bold-type, neighbor-joining in italics, and maximum-likelihood in roman type. Probability values for the fine structure in each clade are not shown for clarity. The scale bar represents amino acid substitutions per site.

**Figure 7:**
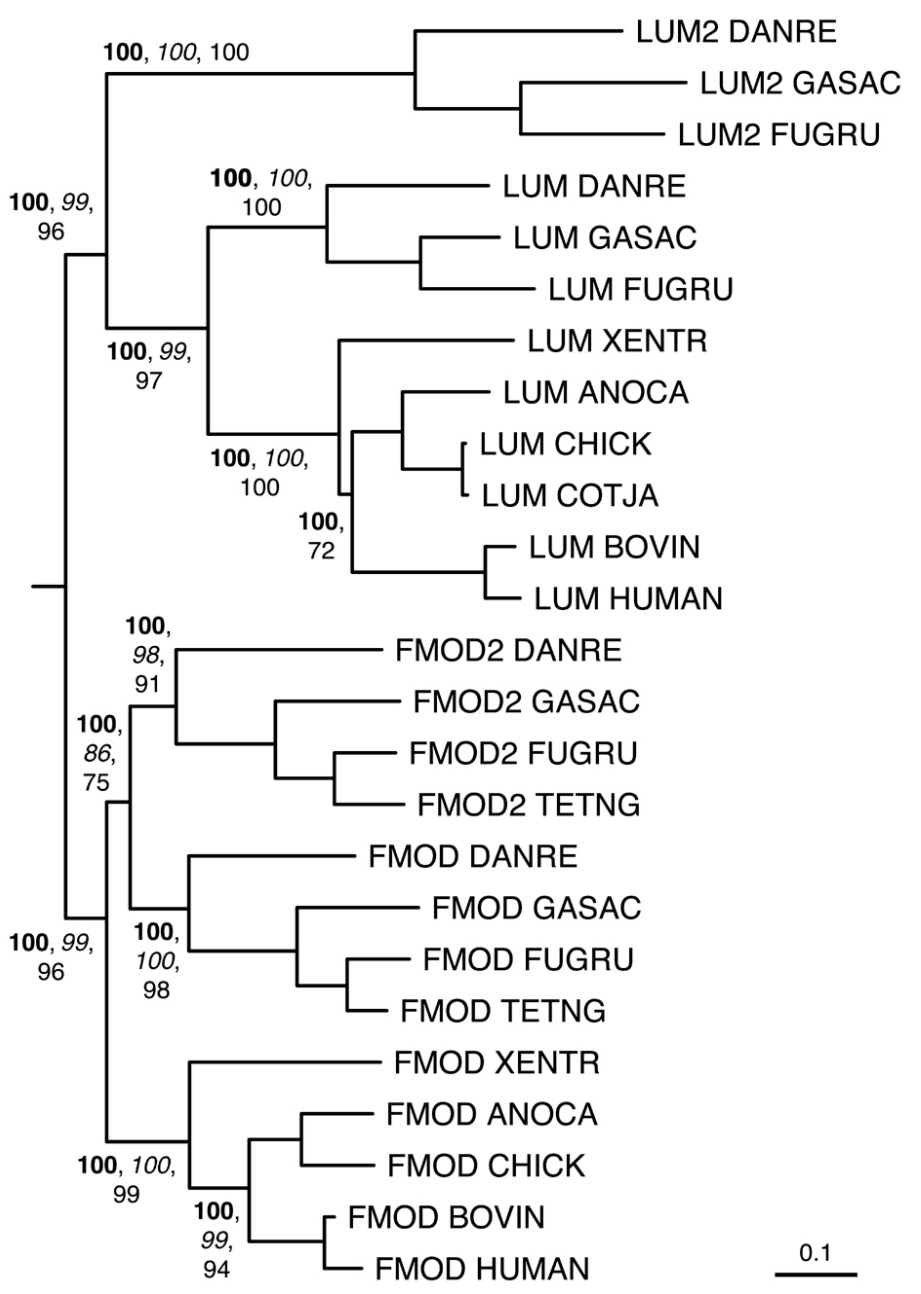
**Phylogenetic relationships of class IIa SLRPs, inferred from the multiple sequence alignment of LRR domains from a reduced set of SLRP sequences (see Methods)**. The tree has been rooted using the midpoint method. Sequences group into two main clusters corresponding to class IIa SLRPs: fibromodulins FMOD and FMOD2, and lumicans LUM and LUM2. The second copies FMOD2 and LUM2 are only present in genomes of ray-finned fishes. Clade probability values higher than 60% are indicated as in Figure 6. The scale bar represents amino acid substitutions per site.

**Figure 8:**
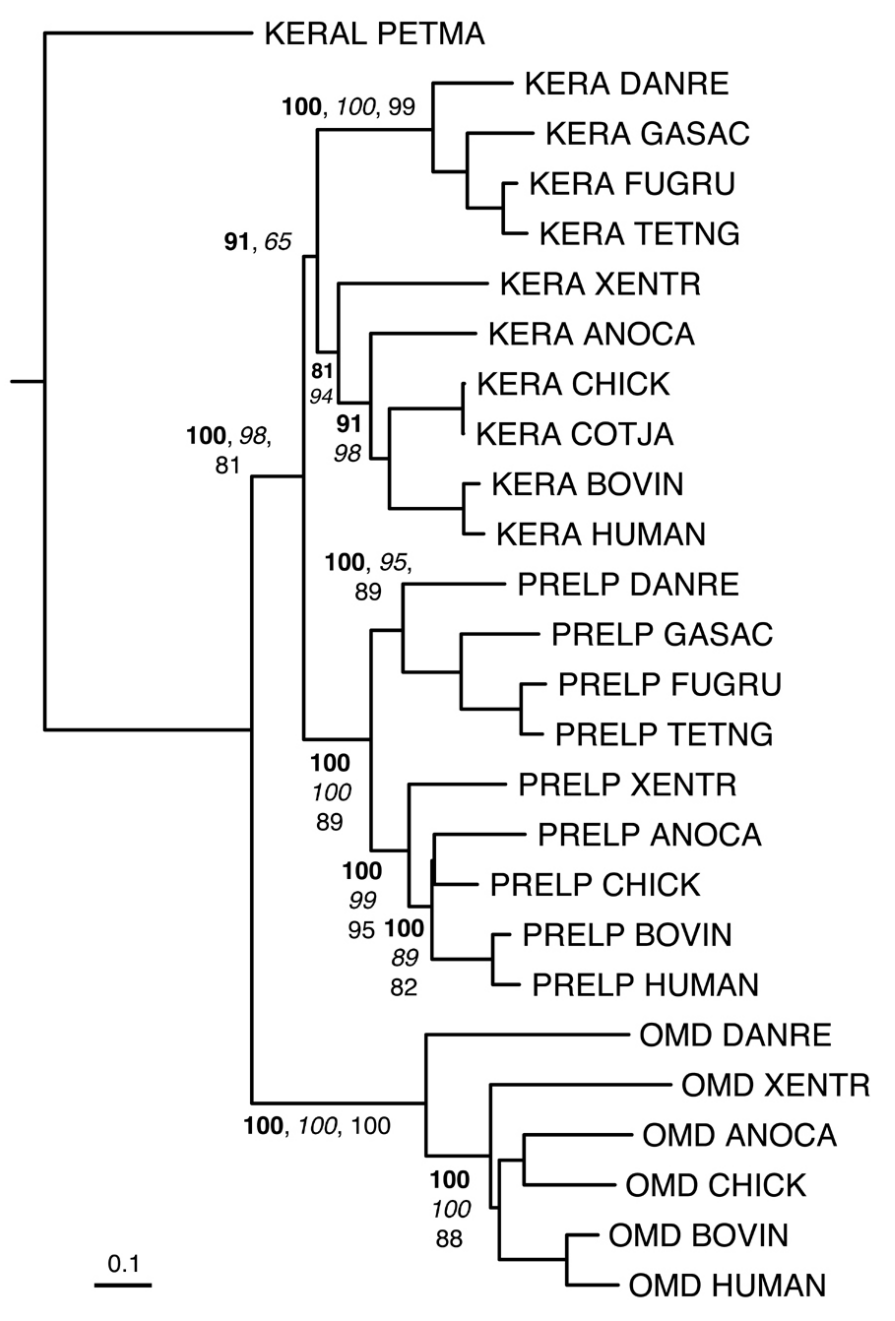
**Phylogenetic relationships of class IIb SLRPs, inferred from the multiple sequence alignment of LRR domains from a reduced set of SLRP sequences (see Methods)**. The tree has been rooted using the lamprey sequence as outgroup. Clade probability values higher than 60% are indicated as in Figure 6. The scale bar represents amino acid substitutions per site.

**Figure 9:**
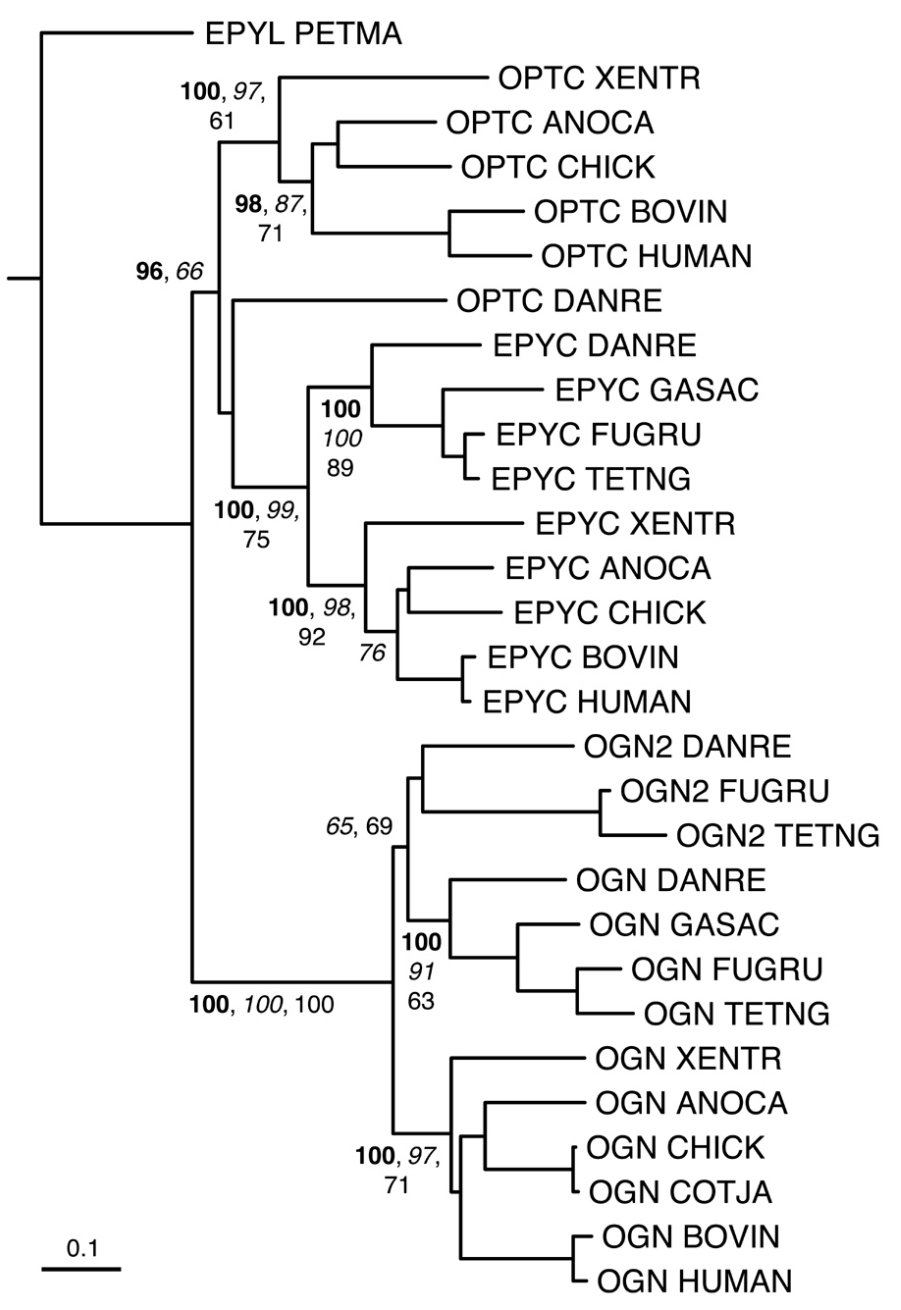
**Phylogenetic relationships of class III SLRPs, inferred from the multiple sequence alignment of LRR domains from a reduced set of SLRP sequences (see Methods)**. The tree has been rooted using the predicted epiphycan-like (EPYL) sequence from lamprey as outgroup. Sequences cluster into three main groups corresponding to class III SLRPs: opticin, epiphycan, and osteoglycins OGN and OGN2. The second copy OGN2 is only present in genomes of ray-finned fishes, whereas the gene for OPTC appears to have largely disappeared from fish genomes, the only known example so far being that of zebrafish. Clade probability values higher than 60% are indicated as in Figure 6. The scale bar represents amino acid substitutions per site.

**Figure 10:**
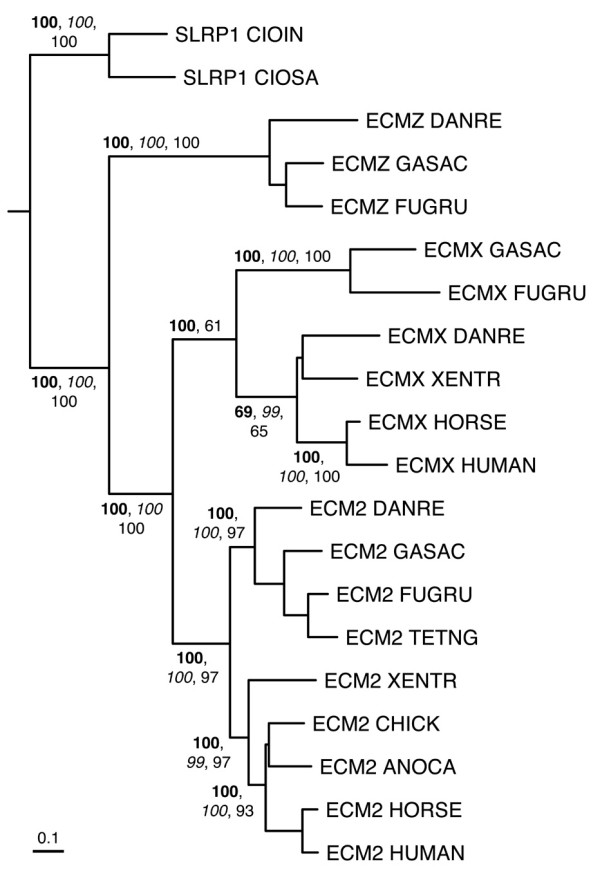
**Phylogenetic relationships of class A SLRPs (ECM2 and ECM2-like sequences), inferred from the multiple sequence alignment of LRR domains from a reduced set of SLRP sequences (see Methods)**. The tree has been rooted using the SLRP1 sequences from the two *Ciona *species as outgroup. Sequences group into three main clusters: ECM2, ECMX (ECM2-like protein from the X chromosome), and ECMZ (ECM2-like predicted protein upstream of the DCN gene in fish genomes). Clade probability values higher than 60% are indicated as in Figure 6. The scale bar represents amino acid substitutions per site.

Figures 6, 7, 8, 9, 10 show partial phylogenetic trees for each SLRP class, inferred from multiple sequence alignments of LRRCE-containing sequences from a subset of genomes (see Methods). Not all SLRP sequences have been found in all genomes. This could be owing to incomplete coverage in some of these genomes or to genuine absence of particular genes due to gene loss. For example, it has been known for some time that chickens do not have a BGN gene [63], and unsurprisingly no such gene has been found in searches against the chicken genome. Interestingly, the BGN gene is present in fishes, reptiles and mammals. The genomes of ray-finned fishes (zebrafish, stickleback, medaka, and the pufferfishes) appear to have an extended set of SLRPs, with additional copies of fibromodulin (FMOD2), lumican (LUM2), osteoglycin (OGN2) or biglycan (BGN2) genes (Figures 6, 7, 8, 9), and yet seem to have mostly lost the genes for OMD and OPTC (with the exception of zebrafish, which appears to have retained a particularly complete set of SLRP genes).

### Newly predicted SLRP sequences related to ECM2 in fishes and mammals

Several ECM2-like genes have also been predicted both in fishes and mammalian genomes (Figures 4 and 10; see also Additional File 1 for sequences and accession codes). A particularly interesting example is that of a protein sequence similar to ECM2 that was first predicted in the genome of zebrafish (*Danio rerio*, accession codes Q1LYN3, XP_690561).

**Figure 11:**
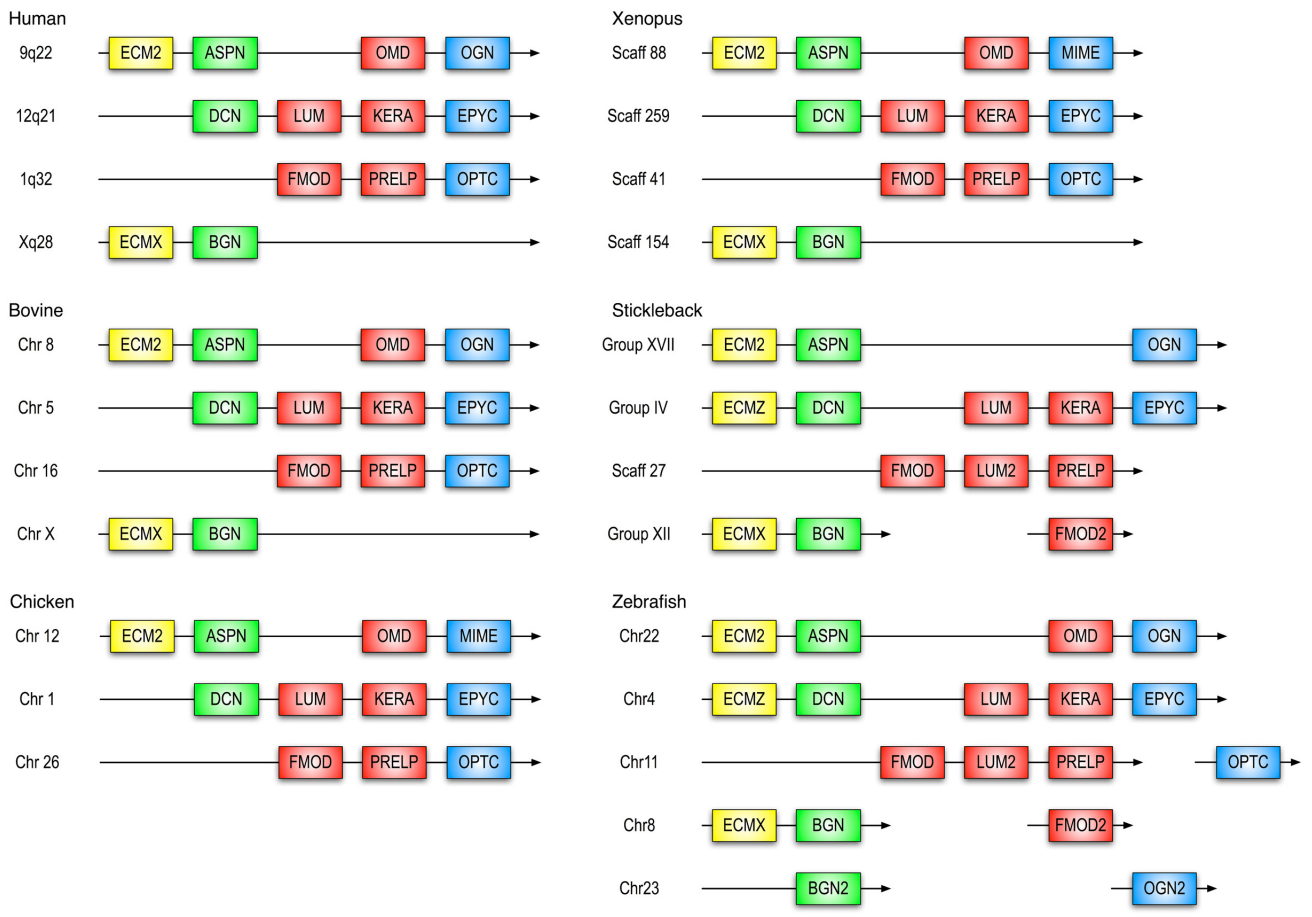
**Synteny of the genes from canonical SLRPs in several vertebrate genomes**. Chromosomal or group location is shown when available in the ENSEMBL database, otherwise scaffold information is provided. Members from the four classes are shown in different colours: yellow (class A), green (class I), red (class II) and blue (class III). Genes shown consecutively do not have any other currently known genes in between, whereas the OPTC, FMOD2 and OGN2 genes in zebrafish and stickleback are separated from the other SLRPs by non-SLRP genes.

This sequence, referred to here as ECMX for reasons explained later, is predicted upstream of the biglycan gene (Figure 11), in a completely analogous manner to the ECM2-ASPN gene tandem seen in mammalian and fish genomes (Figure 11). The predicted ECMX sequence for zebrafish (Table 3) shows a LRR structure highly similar to ECM2, with 15 LRRs and a sequence of repeat lengths highly reminiscent of the 21-24-26 pattern mentioned earlier.

**Table 3:** Two representative examples of ECMX predicted sequences (accession codes in parentheses).

**LRR**	**ECMX_DANRE (Q1LYN3_DANRE)**	***N***	**ECMX_HORSE (XP_001491563)**	***N***
	...HMESLPSGCLLS		...AVPSLPASCLLA	
I	ESLIACGNTRLTQMPIIRDAG	21	RAAIACGNVKMKHVPALTDPG	21
II	VRSLFLADNKISKIPAHALAGLPN	24	LTTLYLAENEIAKIPAHTFLGLPN	24
III	LEWLDLSKNKLDDFSLAPDVFKNLTK	26	LEWLDLSKNKLDAQGLHPHAFKNLTR	26
IV	LRRLNLDGNNFTKVPSLPPS	20	LKRLNLDGNSLSTVPALPTS	20
V	LVELKINDNKLSGLTPHSFKGLAQ	24	LQELKLNDNLLQGLQHSSFQGLSQ	24
VI	LLTLELEDNYFHDGNVSPLAFKPLRQ	26	LLTLEVEGNQLHDGNISPLAFQPLRS	26
VII	LIYLRLDDNKFRAIPSGLPVS	21	LVYLRLDRNQLRTIPPGLPAS	21
VIII	VQELHLSDNKIEVVHSGLLNKTTN	24	LQELHLSTNAIEEVSEGALNRSRN	24
IX	LRVLNLSHNRLREDRIHPRAWIHLLK	26	LRVLVLSNNQLQEDRLAPRAWIDLPK	26
X	LEFLDLSHNKLVHVPSFLPVG	21	LETLDLSHNRLVHVPSFLPRG	21
XI	LRQLVLHHNQIERIPGYVFGHLRPG	25	LRHLTLHHNRIERIPGYVFAHMKPG	25
XII	LDSLQLSYNRLREDGINEVSFIGLYNS	27	LEFLHLSHNSLGADGIHSVSFLGLHAS	27
XIII	LTELLLDHNQLRAIPRGIVQLKS	23	LAELLLDHNQLQAIPRGLLGLRR	23
XIV	LQHLRLNHNYISYVTMNSLCDTTARDDSS	29	LQVLRLSHNKIRYVPLNSICDTRVAQDSN	29
XV	LVSVHLEFNLIERRLIPPTAFSCIRTY	27	LISTHLENNLIDRRRIPPTAFSCIRAY	27
	.QSVLLRPQRYEEHQI		.HSVVLQPQQGEGEGS	

Repeats are numbered in roman numerals, and *N *is the length of each repeat. Sequences amino terminal to the LRRNT cluster have not been included.

Most interestingly, reciprocal interrogation of the human genome using the LRRCE motif from the zebrafish ECMX sequence gave a hit in chromosome X (accession code XP_001714654, currently known as hypothetical protein LOC389904), upstream of the human biglycan gene (Xq28). The predicted protein sequence of this putative novel SLRP is highly similar to the ECMX sequence from zebrafish, although different alternate model assemblies have slightly different number of repeats. This hypothetical protein, which we have named here ECMX owing to its similarity in repeat structure to ECM2 and its location in the X chromosome, has orthologues predicted in the genomes of orangutan, macaque, bovine, horse, dog, opossum (*Monodelphis domestica*), platypus (*Ornithorhynchus anatinus*), frog (*Xenopus tropicalis*), and several fishes (zebrafish, *Gasterosteus aculeatus*, *Oryzias latipes*, *Takifugu rubripes *and *Tetraodon nigroviridis*). The predicted sequence of the horse orthologue (Table 3) maintains the same repeat structure as the zebrafish one. Currently, it is unclear if the differences in repeat structure with the predicted human sequence are significant or if some of the predictions are partially incorrect. Partial transcription evidence for ECMX in humans (EST data) has been obtained from osteoarthritic cartilage and chondrosarcoma (accession codes BQ181183, BQ447619, BQ448435, BQ772123). There is also EST evidence for ECMX in zebrafish (accession codes EB980280, CR929461), *Gasterosteus aculeatus *(DW649744, DW649745), and *Xenopus tropicalis *(CN119819, CX371080, CX409086).

A second ECM2-like predicted sequence occurs upstream of the decorin gene in the genomes of zebrafish, *Gasterosteus aculeatus*, *Oryzias latipes *and *Takifugu rubripes *(Figure 11). This hypothetical protein, which we name here ECMZ, has a predicted sequence that appears to be related to the SLRP1 sequences from both *Ciona *species (Figures 4 and 10). These ECMZ sequences would therefore be the most similar to the ancient SLRP genes (or what is left of them) in vertebrates, and have not been retained in mammals, birds or reptiles. Given the number of ECM2-like sequences and their own clustering with the SLRP1 *Ciona *genes, away from classes I, II and III (Figure 4), we propose a new SLRP class that includes ECM2, ECMX, ECMZ and the *Ciona *SLRP1 sequences. Since these sequences appear to be more closely related to the ancestral gene from which all the canonical SLRPs derived, we name this new class "class A", for ancestral SLRPs. The similarity between the ECM2-ASPN gene tandem and the predicted ECMX-BGN and ECMZ-DCN tandems (Figure 11) strongly supports the notion of SLRP evolution by tandem and large-scale gene duplications as well as tandem gene migration [57,64]. The duplicate copies of SLRP genes exclusive to ray-finned fishes (LUM2, FMOD2, OGN2, BGN2) appear in the same chromosomes as other SLRP sequences (Figure 11). These duplicates may be survivors of the proposed, fish-specific large-scale gene or genome duplication that would have occurred after the divergence between the actinopterygian (ray-finned fishes) and sarcopterygian (coelacanth, lungfish and all land vertebrates) lineages [65-68]. Not all the zebrafish SLRP genes shown in Figure 11 have been identified in the other fish genomes, reflecting the fact that different teleost fishes have retained different sets of duplicate genes [69,70]. Comparison of the zebrafish and fugu genomes have revealed that despite a high degree of synteny and retention of a similar number of duplicates, in a significant number of cases, different paralogues have been preserved [70].

Finally, the chromosomal organization of the canonical SLRP genes in mammals follows the order shown in Figure 11 for the human and bovine genomes, where the class A paralogue is followed downstream by those from classes I, IIa, IIb and III. This organization would suggest that the ECMX-BGN pair might have been initially part of the FMOD-PRELP-OPTC gene cluster that currently is located in the chromosome 1 in humans, and later migrated to the X chromosome (as postulated for the BGN gene in [64]). A hypothetical class IIa gene downstream of ASPN would have disappeared completely, potentially by pseudogenisation. The fish SLRP genes share in part this chromosomal organization (Figure 11), and the class A SLRP upstream of the DCN gene can still be recognized in the predicted ECMZ sequences. An exception is presented by the LUM2 (class IIa) sequence in fishes, which appears intercalated between the FMOD (IIa) and PRELP (IIb) genes but is missing in non-fish genomes. The LUM2 sequence could have originated from additional local gene duplication in fishes or could have migrated from a different SLRP gene cluster. The presence of the additional fish SLRP duplicates (BGN2, FMOD2 and OGN2) points towards a complex history of local and large-scale duplications as well as gene migration for the surviving set of fish SLRPs.

### Possible biological roles for LRRCE motifs

Probably the main role of the LRRCE motifs is to stabilize the LRR structures of the SLRPs by providing a disulphide-bonded C-terminal capping structure, in a similar manner to the LRRCT capping motifs present in many sequences of extracellular or membrane-associated LRR proteins. The requirement of disulphide-bonding integrity for SLRP biological actitvity has been demonstrated for decorin [71] and fibromodulin [72]. Furthermore, thermal denaturation of decorin appears to be completely reversible as long as the disulphide bonds are not reduced [31].

Several diseased states have been associated to mutations occurring in the LRRCE regions of some SLRPs. Two different frame shift mutations on the decorin gene due to single base pair deletions in the LRRCE coding region have been linked to congenital stromal dystrophy of the cornea [73,74]. Both mutations are predicted to result in a truncated decorin protein missing the last 33 C-terminal amino acids. Another mutation resulting in a premature stop codon in the ear extension of keratocan (R313X) has been linked to autosomal recessive cornea plana [75,76]. These two truncations would eliminate most of the LRRCE structure in decorin and keratocan. Three amino acid substitutions in the LRRCE motif of opticin have been linked to high myopia [77], probably through disruption of the local tertiary structure. In all these examples, the predicted truncation or alteration of the LRRCE structure is likely to have a detrimental effect in the stability of the entire LRR domains of these SLRPs.

A direct role of the ear extensions in ligand binding remains an attractive yet still hypothetical scenario. The expanded set of LRRCE sequences presented here shows clear conservation trends across species in the ear extensions of a given SLRP, whereas these extensions are poorly conserved between closely related SLRPs. Thus, the ear extensions could help to differentiate the roles of SLRPs belonging to the same class. In known structures of LRR domains or proteins it is not uncommon to find extended repeats where the polypeptide chain loops out from the expected path of a regular LRR to rejoin it some residues later [5]. These extensions may have functional significance, as shown for example by the so-called β-switch and β-finger in the structure of glycoprotein Ibα [44,45]. Future biochemical and mutagenesis analyses on the SLRP ear extensions will be necessary to elucidate any functional role of these structures in ligand recognition and binding.

## Conclusion

The LRRCE capping motif identified in the structures of the representative SLRPs decorin and biglycan appears to be a structural element exclusive to the main group of SLRPs, which includes the previously described classes I, II and III, plus a new class of ancestral genes that includes ECM2, the SLRP1 sequences from *Ciona*, and other ECM2-like sequences present mainly in fishes but with intriguing orthologues in mammals, including a yet uncharacterized new SLRP in the human X chromosome. The LRRCE motif appears to have evolved during early chordate evolution and is not found in non-chordate LRR protein sequences. Such evolutionary history is probably related to the known interactions of SLRPs with fibrilllar collagens and their regulation of collagen fibrillogenesis [10,33,34]. Given its exclusivity to the SLRP family, the LRRCE motif is a useful annotation tool for the identification and classification of new SLRP sequences in genome sequencing efforts. Analysis of LRRCE-containing sequences of organisms located phylogenetically between critical evolutionary events will provide useful clues for understanding the history of large-scale gene and genome duplications that appear to have occurred during vertebrate evolution. The expanded list of SLRP sequences, provided here for the first time, will facilitate the analysis of residue conservation trends in functionally significant sequence motifs, and ultimately will be useful for the elucidation of the full range of biological functions of this important family of extracellular matrix molecules.

## Methods

### Regular expression pattern and sequence retrieval

An initial set of 58 protein sequences annotated as SLRPs from classes I (21 sequences), II (24 sequences) and III (12 sequences) plus ECM2 (1 sequence) were selected from the Swiss-Prot database (52.1 release, ) and aligned using *CINEMA *[78]. This alignment was used together with the crystal structures of decorin and biglycan (PDB codes 1XKU and 2FT3 respectively), to define a characteristic regular expression pattern using *PROSITE *syntax [79]. This pattern, designated as LRRCE hereupon, was used to retrieve additional sequences from the UniProt database (Swiss-Prot release 55.1, TrEMBL release 38.1) [48] using the ScanProsite tool [80]. The LRRCE regular expression pattern was refined in iterative cycles until no further sequences were obtained. The final LRRCE expression pattern, in *PROSITE *syntax [79], was: [LIV]-X(2)-[LVIYFMA]-X-[LIFM]-X(2)-[NH]-X-[ILVF]-X(2)-[VIMFLY]-X(4)-[FIMLV]-C-X(7,20)-[LYIMV]-X(2)-[ILVTMF]-X-[LVMI]-X(2)-N-X-[IVLMAFT]-X(8,9)-[FYMPVAIS]-X-C.

### Similarity searches and sequence alignment

Additional sequences were obtained through sequence similarity searches (*BLAST *and *TBLASTN*) on the NCBI [81] and ENSEMBL [82] databases. Sequences of LRRCE motifs from different organisms were used to query the different databases. Given the early stages of annotation of some of the genomes, some predicted sequences were manually corrected using supporting genome and EST data. The sequences were aligned using *CLUSTALW *[83] as implemented in the Kyoto University Bioinformatics Center .

### Phylogenetic analyses

The phylogenetic analysis shown in Figure 4 was inferred from a multiple sequence alignment of the LRRCE motifs of the 280 sequences retrieved by ScanProsite and the similarity searches described above. The complete list of sequences used in this study and their accession numbers are provided in Additional File 1. Separate phylogenetic analyses were performed for each SLRP class on a reduced set of sequences from two mammals (human and bovine or horse), two birds (chicken and quail), lizard (anole), frog (*Xenopus*), four teleost fishes (zebrafish, stickleback, fugu and *Tetraodon*), lamprey, and the two *Ciona *species (Figures 6, 7, 8, 9, 10). For each class, phylogenetic analyses were inferred from a gap stripped multiple alignment of the selected sequences generated using *CLUSTALW *[83] and analyzed by three different independent phylogenetic methods. Neighbour-joining (NJ) trees and bootstrap replicates were generated using *SEQBOOT*, *PROTDIST*, *NEIGHBOR *and *CONSENSE *from the *PHYLIP *package [84] using default settings. Maximum Likelihood trees were inferred using *PROML *from the *PHYLIP *package using default settings. Bayesian tree inference values were produced using the MrBayes programme [85], where Markov Chain Monte Carlo analysis was performed for 100,000 generations using 6 chains. Clade-credibility values indicating statistically probable clades (>60%) are indicated from the three methods on the NJ trees (Figures 6, 7, 8, 9, 10), and UniProt organism abbreviations are used for sequence identification: XENTR, *Xenopus tropicalis *(western clawed frog); ANOCA, *Anolis carolinensis *(anole lizard); CHICK, *Gallus gallus *(chicken); COTJA, *Coturnix japonica *(Japanese quail); DANRE, *Danio rerio *(zebrafish); GASAC, *Gasterosteus aculeatus *(stickleback); TETNG, *Tetraodon nigroviridis *(green pufferfish); FUGRU, *Fugu rubripes *(Japanese pufferfish); PETMA, *Petromyzon marinus *(sea lamprey); CIOIN, *Ciona intestinalis *(transparent sea squirt); CIOSA, *Ciona savignyi *(Pacific transparent sea squirt).

### Molecular diagrams

The ribbon diagrams and molecular representations from Figures 1 and 2 were produced using the program *SETOR *[86].

## Authors' contributions

HP is responsible for creating the sequence patterns, data collection and phylogenetic analysis. JHJ contributed to the production and analysis of phylogenetic trees and to the evolutionary and biological insights of the manuscript. RPBH and PNB contributed to the evolutionary and biological insights of the manuscript. TKA contributed to the data analysis and helped in the supervision of the bioinformatic analysis. JB conceived the study, analyzed the data and wrote the manuscript. All authors read and approved the final manuscript.

## Supplementary Material

Additional file 1 **LRRCE sequences and accession codes**. Expanded set of LRRCE sequences including accession codes to sequence databases. Click here for file

Additional file 2 **high-resolution version of Figure **4. Larger version of Figure 4, with legible sequence names at the end of the phylogenetic tree branches. Click here for file
