# Supplementary material for: LRRCE: a leucine-rich repeat cysteine capping motif unique to the chordate lineage
Source: BMC Genomics. 2008 Dec 12;9:599. doi: 10.1186/1471-2164-9-599 (PMC2637281; doi:10.1186/1471-2164-9-599)
Supplement: Additional file 1 — LRRCE sequences and accession codes. Expanded set of LRRCE sequences including accession codes to sequence databases. [file 1471-2164-9-599-S1.pdf]

Expanded set of LRRCE sequences. Accession codes are as follows: SwissProt in blue, TrEMBL in red, NCBI in purple, and ENSEMBL in green. The residue colour scheme is as in Figure 3. UniProt taxonomic abbreviations are used in this list: HUMAN, *Homo sapiens*; PANTR, *Pan troglodytes* (chimpanzee); PONPY, *Pongo pygmaeus* (orangutan); MACMU, *Macaca mulatta* (rhesus macaque); MACFA, *Macaca fascicularis* (crab eating macaque); BOVIN, *Bos taurus* (bovine); HORSE, *Equus caballus*; SHEEP, *Ovis aries*; CAPHI, *Capra hircus* (goat); PIG, *Sus scrofa*; CANFA, *Canis familiaris* (dog); MOUSE, *Mus musculus*; RAT, *Rattus norvegicus*; RABIT, *Oryctolagus cuniculus* (rabbit); DASNO, *Dasypus novemcinctus* (armadillo); MONDO, *Monodelphis domestica* (opossum); ORNAN, *Ornithorhynchus anatinus* (platypus); XENLA, *Xenopus laevis* (African clawed frog); XENTR, *Xenopus tropicalis* (western clawed frog); ANOCA, *Anolis carolinensis* (anole lizard); CHICK, *Gallus gallus* (chicken); COTJA, *Coturnix japonica* (Japanese quail); DANRE, *Danio rerio* (zebrafish); GASAC, *Gasterosteus aculeatus* (stickleback); ORYLA, *Oryzias latipes* (medaka fish); SALSA, *Salmo salar* (Atlantic salmon); ORENI, *Oreochromis niloticus* (Nile tilapia); PAROL, *Paralichthys olivaceus* (Japanese flounder); FUGRU, *Fugu rubripes* (Japanese pufferfish); TETNG, *Tetraodon nigroviridis* (green pufferfish); PETMA, *Petromyzon marinus* (sea lamprey); CIOIN, *Ciona intestinalis* (transparent sea squirt); CIOSA, *Ciona savignyi* (Pacific transparent sea squirt).

Class A: ECM2 (Extracellular matrix protein 2); ECMX (ECM2-like protein from the X chromosome); ECMZ (ECM2-like predicted protein upstream of the DCN gene in fish genomes); SLRP1 (the small leucine-rich repeat protein from *Ciona intestinalis* and *Ciona savignyi*).

ECM2_HUMAN	O94769	LHFLRLNNNKIRNILPEEICNAEEDDD........SN...LEHLHLENNYIKIREIPSYTFSCIRSYSSIVLKPQNIK
ECM2_PANTR	ENSPTRG00000021114	LHFLRLNNNKIRNILPEEICNAEEDDD........SN...LEHLHLENNYIKIREIPSYTFSCIRSYSSIVLKPQNIK
ECM2_PONPY	ENSPPYG00000019380	LHFLRLNNNKIRNILPEEICNAEEDDD........SN...LEHLHLENNYIKIREIPSYTFSCIRSYSSIVLKPQNIK
ECM2_MACMU	ENSMMUG00000004640	LHFLRLNNNKIRNILPEEICNAEEDDD........SN...LEHLHLENNYIKIREIPSYTFSCIRSYSSIVLKPQNIK
ECM2_BOVIN	Q3MHH9	LHFLRLNNNKIRNILPEQICNAEEDDD........SN...LQHLHLENNYIKTREIPSYAFSCIRSYSSIVLKPQNIK
ECM2_HORSE	XP_001491707	LHFLRLNNNKIRTILPEQICNAEEDDD........ST...LEHLHLENNYIKTREISPYAFSCIRYYSSIVLKPQNIK
ECM2_CANFA	XP_533562	LHFLRLNNNKIRNILPEQICNAEEDGD........ST...LEHLHLENNYIKTREISSYAFSCIRSYSSIVLKPQNIK
ECM2_MOUSE	Q5FW85	LHFLRLNNNKIRNIHPEQICNAEEDED........SA...LEHLHLENNYIRTREISSYAFSCIRLYSSIVLKPQHIK
ECM2_RAT	ENSRNOG00000031716	LHFLRLNNNKIRNILPEQICNAEEDED........SA...LEHLHLENNYITTRAMSPYAFSCIRLYSSVVLKPQRIK
ECM2_RABIT	ENSOCUG00000008288	LHFLRLNNNKIRNILPEQVCIAEEDGD........SM...LEHLHLENNYIKTREIPSYAFSCIRSYSSIVLKPQKVK
ECM2_MONDO	XP_001371338	LHLLRLNNNKIRNVLPQRVCRAKGRD.........SP...LEQLHLENNYIRLRDLPLHLFSCLRSYASIVLRPQKAK
ECM2_ORNAN	XP_001521514	LHFLRLNDNKIRSVPPERICNSEDDGD........SS...LEHLHLENNYINTREISPYAFSCIRSYSSIVLKPQKIK
ECM2_XENTR	ENSXETG00000020918	LHILRLNNNKIRSVSPYCICSAEDEGD........SS...IEQLHLENNLINTREISPYAFFCIRSYSSVILKPQKVK
ECM2_ANOCA	GENSCAN00000110086	LNFLRLNNNKIRAVPTERICRIRRDNEDDEEE<br>DDSEEDHDYED....SQ...LEHLHLEYNYINTRDLSPYAFSCVRSYSSVILKPQKIK
ECM2_CHICK	XP_414297	LRFLRLNNNKIRTVPPERICRTHLHDDDVHY<br>NSEEDEGED......SR...LEHVHLENNYINTRQLSPHSFSCIRSYCSVVLKPQKTK
ECM2_DANRE	Q1LXA8	LHFLRLNNNKIRHVPEQAICDPLSEDD........SH...LVAVRLENNFIDPRKIPPTAFSCVRSYSSVVLKPQRIK
ECM2_GASAC	ENSGACG00000004058	LHFLRLNDNDIRSVPDEGICDPHHSGD........ST...LVAVRLENNRLDPQKISPTAFSCVRSSSSVVLKPQKTK
ECM2_ORYLA	ENSORLG00000019864	LHFLRLDNNMIRSIPEDSICDPDSNGD........TS...LVALRLENNFINSNKISPTAFSCIRAASSVVLKPQKVKN
ECM2_FUGRU	ENSTRUG00000009473	LHFLRLNNNKIRRVPEESICDPDKDGD........PN...LVVLRLENNYIDSKTISPTAFSCVHATSSVILKPQKTK
ECM2_TETNG	Q4TBJ8	LHFLRLNNNQIRSVPEESICDPDREGD........PN...LVVLRLENNYIDSKKISPSAFSCVHAPSSVILKPQKTK

ECMX_HUMAN	XP_001714654	LQVLGLSHNRIRQVPLNSICDMRVAQD........SN...LTSTHLENNLIDRRRIPPTAFSCTRAYHSVVLQPQRRGEEGS
ECMX_PANTR	GENSCAN00000055668	LQVLGLSHNRIRQVPLNSICDTRVAQD........SN...LISTHLENNLFDRRRIPPTAFSCIRAYHSVVLQPQRGEEEGS
ECMX_PONPY	GENSCAN00000000880	LQVPGLSHNRIRQVPLNSICDTRVAQD........SN...LISTHLENNLIDRHRIPPTAFSCIRAYHSVVLQPQRGEEKGS
ECMX_MACMU	GENSCAN00000111935	LQVLGLSHNRIRQVPLNSICDMHMAQD........SN...LISIHLENNLTDQRRIPPTAFSCIRAYHSVVLQPQRGEEEGS
ECMX_BOVIN	XP_596290	LQVLRLSHNKIRHVPLNSICDTRVAQD........SN...LISTHLENNLIDRRRIPPTAFSCIRAYHSVVLEPQQGEGESA
ECMX_HORSE	XP_001491563	LQVLRLSHNKIRYVPLNSICDTRVAQD........SN...LISTHLENNLIDRRRIPPTAFSCIRAYHSVVLQPQQGEGEGS
ECMX_MONDO	XP_001377851	LQVLRLSHNKIRHVPLNSVCDTRVAQD........SN...LISMHLENNLIDRRRIPPTAFSCIKAYHSVVLKPQQDEEEY
ECMX_ORNAN	XP_001510778	LQVLRLSHNKIRHVPLNSICDTRVAED........SN...LISMHLENNLIDRRRIPPTAFSCIKAYHSVVLRPQQNEEDY
ECMX_XENTR	ENSXETESTG00000006397	LQVLRLSFNKIRHVPLNSICDTRVSVD........SN...LVSVHLENNFINRRLIPPTAFSCIKTYHSVVLRPQHGEYEYY
ECMX_DANRE	Q1LYN3	LQHLRLNHNYISYVTMNSLCDTTARDD........SS...LVSVHLEFNLIERRLIPPTAFSCIRTYQSVLLRPQRYE
ECMX_GASAC	ENSGACG00000010076	LQVLRLDHNQIRLVRQWGVCHPRSSG.........SS...LASIHLENNLLDVERIPPKAFSCLTDAQGLILYPQQRLSHDQ
ECMX_ORYLA	ENSORLG00000008687	LQMLRLDNNKIRLMRPWGVCHPRNSG.........S....LASVHLENNLLEVETLPPKAFSCVIDAKGLILYPQQYKKDENT
ECMX_FUGRU	ENSTRUG00000009797	LKMLRLDNNRIRAVRRWGICHPRNSG.........SM...LAAVHLENNRLQVDRIPADTFSCLIDAQGLVLYPQQGQ

ECMZ_DANRE	GENSCAN00000040711	LHSATFMHNNIRSIPRDAFCWGRDNVSP......LSR...LVKVQLEYNMIDLGHLDTLAFRCLRGFQVVELY
ECMZ_GASAC	GENSCAN00000025147	LRTVALNHNRIKSVPRDAFCWGNKSVS.......LSG...LVRVQLEHNLIDVGKLDAQAFRCLRGFQVVHFY
ECMZ_ORYLA	GENSCAN00000007987	LHSVALSHNRIQSVPHDAFCWGNKT.........LSG...LVRVQLEHNFIDFGEMDARAFSCLRGSQVVHFY
ECMZ_FUGRU	ENSTRUG00000013884	LHSVALTHNRIQSVPSDAFCWGDNH.........LSR...LVRVQLEDNLIDVGKLNTQAFRCLRGFQVVHLY
SLRP1_CIOIN	ENSCING00000012194	LEALFLESNQIFSVQIDSFCPMNPERFV......TSK...LHTLRLDGNLIDSNDLPALAFYCL.SSMEVVVT
SLRP1_CIOSA	SINCSAVG00000002276	LEALFLESNQIFSVQIDSFCPMNPDRFV......TSK...LHTLRLDDNLIEPGSIPALAFYCL.LSMEAVVT

Class I: ASPN (asporin); BGN and BGN2 (biglycans); DCN (decorin); BGL1 and BGL2 (biglycan-like sequences in lamprey); DCL1 and DCL2 (decorin-like sequences in lamprey)

ASPN_HUMAN	Q9BXN1	LQIIFLHSNSIARVGVNDFCPTVPKMK.......KSL...YSAISLFNNPVKYWEMQPATFRCVLSRMSVQLGNFGM
ASPN_PANTR	XP_001146178	LQIIFLHSNSIARVGVNDFCPTVPKMK.......KSL...YSAISLFNNPVKYWEMQPATFRCVLSRMSVQLGNFGM
ASPN_PONPY	ENSPPYG00000019379	LQIIFLHSNSIARVGVNDFCPTVPKMK.......KSL...YSAISLFNNPVKYWEMQPATFRCVLSRMSVQLGNFGM
ASPN_MACMU	ENSMMUG00000004638	LQIIFLHSNSIARVGVNDFCPTVPKMK.......KSL...YSAISLFNNPVKYWEMQPATFRCVLSRMSVQLGNFGM
ASPN_MACFA	BAE89770	LQIIFLHSNSIARVGVNDFCPTVPKMK.......KSL...YSAISLFNNPVKYWEMQPATFRCVLSRMSVQLGNFGM
ASPN_BOVIN	Q3ZBN5	LQIIFLHSNSITKVGVNDFCPTVPKMK.......KSL...YSAISLSNNPVKYWEVQPATFRCVLSRMSVQLGNFRK
ASPN_HORSE	XP_001146178	LQIIFLHSNSITKVGVNDFCPTVPKMK.......KSL...YSAISLFNNPVKYWEVQPATFRCVLSRMSVQLGNFRK
ASPN_CANFA	XP_853321	LQIIFLHSNSITKVGVNDFCPTVPKMK.......KSL...YSAISLSNNPVRYWEVQPATFRCVVSRMSVQLGNFRK
ASPN_MOUSE	Q99MQ4	LQIIFLHYNSIAKVGVNDFCPTVPKMK.......KSL...YSAISLFNNPMKYWEIQPATFRCVLGRMSVQLGNVGK
ASPN_RAT	Q5XIH1	LQIIFLHNNSITKVGVNDFCPTVPKMK.......KSL...YSAISLFNNPMKYWEIQPATFRCVVGRMSVQLGNVGK
ASPN_DASNO	ENSDNOG00000012510	LQIIFLHSNSITKVGVNDFCPTVPKMK.......KSL...YSAISLFNNPVKYWEVQPATFRCVLNRMSVQLGNFRK
ASPN_ORNAN	XP_001516270	LQVIFLHSNSISKLGVNDFCPTGTKMK.......KSL...YSAISLFNNPVKYWEVQPATFRCVLSRMSVQLGNFRK
ASPN_XENLA	Q5XHD6	LQVLFLHSNNIGKVDVNDFCPTGLKLK.......KSL...YSGISLFKNPVKYWEVQPATFRCTLGRNSVHLGNFRK
ASPN_XENTR	ENSXETG00000027246	LQVLFLHSNNIGKVDVNDFCPTGLKLK.......KSL...YSGISLFKNPVKYWEVQPATFRCTLGRNSVHLGNFRK

ASPN_ANOCA	GENSCAN00000066137	LQVAFLHSNSISKVGVNDFCPTGGRLK.......KTL...YSGISLFNNPVKYWDIQPGTFRCILNRNSVQIGNYGK
ASPN_CHCK	XP_414298	LQVVFLHSNHIAKLGVNDFCPTGRRKK.......KAL...YSGISLFNNPVKYWEVQPSTFRCILARNSVQLGNFLK
ASPN_DANRE	Q1LXA7	LQVMYLHANSIGYVGVNDFCPSRTRAK.......KAL...YTRISLYANPVKYWEIQPPTFRCVSSHNSVQLGNHRK
ASPN_GASAC	ENSGACG00000004064	LQVIYLHGNKISGVGVNDFCPVPGGVK.......KNP...YTGISLFANPVKYWDVQPAAFRCVSGRRGVQMGNFRK
ASPN_ORYLA	ENSORLG00000019874	LQVIFLHNNKISYVGMNDFCPSSTGGK.......KNP...YTGISLFANPVKYWNIQPAIFRCVTGRRAVHLGNFRK
ASPN_ORENI	Q9DE04	LQVIFLHGNKISSVGINDFCPIRADSK.......KNP...YTGISLFANPVKYWAIQPATFRCVTGRRGVQLGNFRK
ASPN_FUGRU	ENSTRUG00000009473	LQVIFLHGNKISKVGVNDFCPINPSMK.......KNK...YAIISLFANPVKYWEVNPATFRCVTGRRSIQLGNFKK
ASPN_TETNG	Q4TBJ7	LQVIFLHGNKISKVGVNDFCPINPSTK.......KTR...YSIISLFGNPVKYWEVNPVTFRCVTGRRSIHLGNFK
BGN_HUMAN	P21810	LQVVYLHSNNITKVGVNDFCPMGFGVK.......RAY...YNGISLFNNPVPYWEVQPATFRCVTDRLAIQFGNYKK
BGN_PONPY	Q5RAY4	LQVVYLHSNNITKVGVNDFCPVGFGVK.......RAY...YNGISLFNNPVPYWEVQPATFRCVTDRLAIQFGNYKK
BGN_MACMU	ENSMMUG00000012743	LQVVYLHSNNITKVGVNDFCPVGFGVK.......RAY...YNGISLFNNPVPYWEVQPATFRCVTDRLAIQFGNYKK
BGN_BOVIN	P21809	LQVVYLHTNNITKVGVNDFCPVGFGVK.......RAY...YNGISLFNNPVPYWEVQPATFRCVTDRLAIQFGNYKK
BGN_HORSE	O46403	LQVVYLHTNNITKVGVNDFCPVGFGVK.......RAY...YNGISLFNNPVPYWEVQPATFRCVTDRLAIQFGNYKK
BGN_SHEEP	O46390	LQVVYLHTNNITKVGVNDFCPVGFGVK.......RAY...YNGISLFNNPVPYWEVQPATFRCVTDRLAIQFGNYKK
BGN_CANFA	O02678	LQVVYLHTNNITKVGVNDFCPVGFGVK.......RAY...YNGISLFNNPVPYWEVQPATFRCVTDRLAIQFGNYKK
BGN_MOUSE	P28653	LQVVYLHSNNITKVGINDFCPMGFGVK.......RAY...YNGISLFNNPVPYWEVQPATFRCVTDRLAIQFGNYKK
BGN_RAT	P47853	LQVVYLHSNNITKVGINDFCPMGFGVK.......RAY...YNGISLFNNPVPYWEVQPATFRCVTDRLAIQFGNYKK
BGN_ORNAN	XP_001510892	LQVVYLHSNNITKVGVNDFCPIGFGVK.......RAY...YNGISLFNNPVPYWEVQPATFRCVTDRLAIQFGNYKK
BGN_XENLA	Q9IB75	LQVVYLHSNNITQVGVNDFCPIGFGVK.......RAY...YNGISLFNNPVPYWEVQPATFRCVTDRLAIQFGNYRK
BGN_XENTR	Q0P4I3	LQVVYLHSNNITQVGVNDFCPVGFGVK.......RTY...YNGISLFNNPVPYWEVQPATFRCVTDRLAIQFGNYRK
BGN_ANOCA	GENSCAN00000050413	LQVVYLHSNNITHIGVNDFCPVGFGVK.......RAS...YHGISLFANPVPYWEVQPATFRCATDRLAIQFGNYKK
BGN_DANRE	Q6GMI5	LQVVYLHSNNITNVGEDDFCPTGFGLK.......KVF...YNGISLFDNPIRYWEVQPSTFRCVSDQMAVQFGNHKK
BGN_GASAC	ENSGACG00000010078	LQVVYLHSNAIDQVGVDDFCPRGFGMK.......RTF...YNGISLFSNPVNYWEVQPATFRCVGNRLGIQFGNYKKK
BGN_ORYLA	ENSORLG00000008709	LQVVYLHSNSINHVDVDDFCPRGFGMK.......RTF...YNGISLFDNPVNYWDVQPATFRCVNDRYAIQFGNYKK
BGN_FUGRU	ENSTRUG00000009612	LQVVYLHSNNIDQVGVNDFCPRGFGMK.......RVF...YNGISLFSNPVNYWEVQPATFRCVTDRLAIQFGNYKK
BGN_TETNG	Q4T5R8	LQVVYLHSNNIDHVGVNDFCPRGFGMK.......RVF...YNGISLFSNPVNYWEVQPATFRCVTDRLAIQFGNYKK
BGN2_DANRE	XP_001330860	LQVAYLHSNNISQVDVNDFCPQGFGMK.......RNF...YHGISLYGNPVNYWEVQPATFRCVADRLAIQFGNYKK
DCN_HUMAN	P07585	IQVVYLHNNNISVVGSSDFCPPGHNTK.......KAS...YSGVSLFSNPVQYWEIQPSTFRCVYVRSAIQLGNYK
DCN_PANTR	Q5R1V9	IQVVYLHNNNISVVGSSDFCPPGHNTK.......KAS...YSGVSLFSNPVQYWEIQPSTFRCVYVRSAIQLGNYK
DCN_PONPY	ENSPPYG00000004826	IQVVYLHNNNISVVGSSDFCPPGHNTK.......KAS...YSGVSLFSNPVQYWEIQPSTFRCVYVRSAIQLGNYK
DCN_MACMU	ENSMMUG00000016997	IQVVYLHNNNISVVGSSDFCPPGHNTK.......KAS...YSGVSLFSNPVQYWEIQPSTFRCVYVRSAIQLGNYK
DCN_MACFA	Q4R5D2	IQVVYLHNNNISVVGSSDFCPPGHNTK.......KAS...YSGVSLFSNPVQYWEIQPSTFRCVYVRSAIQLGNYK
DCN_BOVIN	P21793	IQVVYLHNNNISAIGSNDFCPPGYNTK.......KAS...YSGVSLFSNPVQYWEIQPSTFRCVYVRAAVQLGNYK
DCN_HORSE	O46542	IQVVYLHNNNISAVGSNDFCPPGYNTK.......KAS...YSGVSLFSNPVQYWEIQPSTFRCVYVRSAIQLGNYK
DCN_SHEEP	Q9TTE2	IQVVYLHNNNISAIGSNDFCPPGYNTK.......KAS...YSGVSLFSNPVQYWEIQPSTFRCVYVRAAVQLGNYK
DCN_PIG	Q9XSD9	IQVVYLHNNNISAVGSNDFCPPGYNTK.......KAS...YSGVSLFSNPVQYWEIQPSTFRCVYVRSAIQLGNYK
DCN_CANFA	Q29393	IQVVYLHNNNISAVGSNDFCPPGYNTK.......KAS...YSGVSLFSNPVQYWEIQPSTFRCVYVRSAIQLGNYK

DCN_MOUSE	P28654	IQVVYLHNNNISAVGQNDFCRAGHPSR.......KAS...YSAVSLYGNPVRYWEIFPNTFRCVYVRSAIQLGNYK
DCN_RAT	Q01129	VQVVYLHNNNISEVGQHDFCLPSYQTR.......KTS...YTAVSLYSNPVRYWQIHPHTFRCVFGRSTIQLGNYK
DCN_RABIT	Q28888	IQVVYLHNNNISVVGANDFCPPGYNTK.......KAS...YSGVSLFSNPVQYWEIQPSTFRCVYMRSAIQLGNYK
DCN_MONDO	XP_001363160	IQVVYLHNNNISNVGSNDFCPPGYNTK.......KAS...YSGVSLFSNPVRYWEIQPSTFRCVYERSAIQLGNYK
DCN_ORNAN	XP_001511900	IQVVYLHNNNISAVGSNDFCPPGYNTK.......KAS...YSGVSLFSNPVKHWEILPSTFRCVYERSAIQLGNFK
DCN_XENLA	Q66J59	IQVVYLHNNKIAAVSTNDFCPLGYNTK.......KAS...YTGISLFSNPVQYWEIQPATFRCVYERSAIQIGNYK
DCN_XENTR	A4IIC2	IQVVYLHNNKISAVSTNDFCPLGYNTK.......KAS...YSGISLFSNPVQYWEIQPATFRCVYERSAIQIGNYK
DCN_ANOCA	GENSCAN00000094136	IQVVYLHNNKIASIGINDFCPLGYNTK.......KAT...YSGVSLFSNPVQYWEIQPSAFRCIHERSAVQIGNYK
DCN_CHICK	P28675	IQVVYLHNNKIASIGINDFCPLGYNTK.......KAT...YSGVSLFSNPVQYWEIQPSAFRCIHERSAVQIGNYK
DCN_COTJA	Q9DE68	IQVVYLHNNKIASIGINDFCPLGYNTK.......KAT...YSGVSLFSNPVQYWEIQPSAFRCIHERSAVQIGNYK
DCN_DANRE	Q5RI45	IQVIYLHSNKIAAVGTEDFCPPGYNTK.......KAM...YSGISLFSNPVPYWEVQPITFRCVFDRSAIQLGNYRKK
DCN_GASAC	ENSGACG00000019806	IQVVYLHANKIAAVGTEDFCPPGLNQK.......KAM...YSGISLFSNPVPYWEIPPITFRCVFDRSAIQLGNYRKK
DCN_ORYLA	ENSORLG00000015308	IQVVYLHSNKIAAVGTEDFCPPNFNTK.......KAM...YSGISLFSNPVPYWEVQPITFRCVFDRTAIQLGNYR
DCN_SALSA	Q0ZHF2	IQVIYLHSNKIGVVGTQDFCPPGYNTK.......KAM...YSGISLFSNPVPYWEVQPITFRCVFDRSAIQLGNYRKK
DCN_PAROL	Q6J0Y6	IQVVYLHVNKIAAVGTEDFCPPGFNTK.......KAM...YSGISLFSNPVPYWEVQPITFRCVFDRSAIQLGNYRKK
DCN_ORENI	Q9DE03	IQVVYLHAHKIAAVGTEDFCPPGFNTK.......KAM...YSGISLFSNPVPYWEVQPVTFRCVFDRSAIQLGNYRKK
DCN_FUGRU	ENSTRUG00000013771	IQVVYLHVNKIAAVGTGDFCPPSINSK.......KAM...YSGISLFSNPVPYWEVQPTTFRCVFQRSAIQLGNYRKK
DCN_TETNG	GSTENG00020802001	IQVVYLHAKKIAAVGTEDFCPPSINSK.......KAM...YSGISLFSNPVPYWEVQPITFRCVFQRSAIQLGNYRKK
BGL1_PETMA	Q9DDZ7	LQVVYLHSNKIAAVKSDDFCSKGASPK.......RVL...YSGISLFDNPVNYWDVPPSAFRCVASRSAVQFSQNFRK
BGL2_PETMA	Q9DDZ9	LNVVYLHSNKIKEVKPTDFCPTVFSPK.......RAQ...YAGISLYDNPVKYWEVPPSVFRCVHNHNAIHFGSNYRK
DCL1_PETMA	GENSCAN00000064412	IMAVYLHENKISVVEPDDFCGKGHSPK.......KSL...YSAISLYRNPVNYWDIQPLAFRCVTSRVAVQLGNYRK
DCL2_PETMA	GENSCAN00000041728	XXVIFLHDNKIASIFNDSFCPPVFNPK.......KAL...YTSISLFGNALRPWEVPASAFSCVSGRAALHLGNYRK

Class II: LUM and LUM2 (lumicans); FMOD and FMOD2 (fibromodulins); KERA (keratocan); PRELP (prolargin); OMD (osteomodulin); KERAL (keratocan-like sequence from lamprey)

LUM_HUMAN	P51884	LENYYLEVNQLEKFDIKSFCKILGPLS.......YSK...IKHLRLDGNRISETSLPPDMYECLRVANEVTLN
LUM_PANTR	XP_001135206	LENYYLEVNQLEKFDVKSFCKILGPLS.......YSK...IKHLRLDGNRISETSLPPDMYECLRVANEVTLN
LUM_PONPY	Q5RFG1	LENYYLEVNQLEKFDVKSFCKILGPLS.......YSK...IKHLRLDGNRISETSLPPDMYECLRVANEVTLN
LUM_MACMU	ENSMMUG00000016995	LENYYLEVNQLEKFDVKSFCKILGPLS.......YSK...IKHLRLDGNRISETSLPPDMYECLRVANEVTLN
LUM_MACFA	Q4R4R3	LENYYLEVNQLEKFDVKSFCKILGPLS.......YSK...IKHLRLDGNRISETSLPPDMYECLRVANEVTLN
LUM_BOVIN	Q05443	LENYYLEVNELEKFDVKSFCKILGPLS.......YSK...IKHLRLDGNHITQTSLPPDMYECLRVANEITVN
LUM_HORSE	A2Q0Z2	LENYYLEVNELEKFEVKSFCKILGPLS.......YSK...IKHLRLDGNRLTHTSLPPDMYECLRVANEITVT
LUM_CANFA	XP_539716	LENYYLEVNELEKFEVKSFCKILGPLS.......YSK...IKHLRLDGNRLTHTSLPPDMYECLRVANEITVN
LUM_MOUSE	P51885	LENYYLEVNELEKFDVKSFCKILGPLS.......YSK...IKHLRLDGNPLTQSSLPPDMYECLRVANEITVN
LUM_RAT	P51886	LENYYLEVNKLEKFDVKSFCKILGPLS.......YSK...IKHLRLDGNPLTQSSLPPDMYECLRVANEITVN
LUM_RABIT	ENSOCUG00000005592	LENYYLEVNELEKFEVKSFCKILGPLS.......YSK...IKHLRLDGNHLTQMSLPPDMYECLRVANEITVN

LUM_DASNO	ENSDNOG00000018904	LENYYLEVNELDKFEVRSFCKILGPLS.......YSK...IKHLRLDGNRLTQNSLPPEMYECLRVANDITVNVN
LUM_MONDO	XP_001368917	LENYYLEVNEIDKFEVKSFCRVLGPLS.......YSR...VKHLRLDGNLLSHSKLPSEIYDCLRVANDITLE
LUM_ORNAN	XP_001510928	LENYYLEVNEIEKFAVSSFCKVLGPLS.......YSK...VKHLRLDGNKIKRSNLPPEMYECLRVANDIALE
LUM_XENLA	Q7SYS8	LENLYMQVNKIQKFTLNSFCKVIGPLE.......YSK...IRHLRLDGNNISRIDLPQDMYSCLRVASEIDLG
LUM_XENTR	Q640B1	LENLYLQVNKIQKFNLNSFCKIIGPLE.......YSK...IRHLRLDGNNISRVDLPQDMYSCLRVASEIDLG
LUM_ANOCA	GENSCAN00000094136	LENYYLQVNQINKFPVSSFCKVVGALA.......YSR...VRHLRLDANNLTRSDLPQEMYNCLRMAADIAL
LUM_CHICK	P51890	LENFYLQVNKINKFPLSSFCKVVGPLT.......YSK...ITHLRLDGNNLTRADLPQEMYNCLRVAADISLE
LUM_COTJA	Q9DE67	LENFYLQVNKINKFPLSSFCKVVGPLT.......YSK...ITHLRLDGNNLTRADLPQEMYNCLRVAAEISLE
LUM_DANRE	Q6IQQ7	LEHLYLQVNEINKFELTNICRFSSPVN.......YSR...LRTLRLDGNNITHSSMPDDTANCLRQASEIIFE
LUM_GASAC	ENSGACG00000019814	LQQLYLQANEINKFELTSFCKLVTTVN.......FSH...LKHLRLDANQITYSSMPPEYSNCLRHASEIIFE
LUM_ORYLA	ENSORLG00000015318	LEQLYLQANEINKFDLSSFCKYTSPLN.......YSR...LKHLRLDANNITHGSMPPETSNCLRVASDVMFE
LUM_FUGRU	GENSCAN00000027077	LEHLYLQANEINKFDLSSFCKYVTPTD.......FSH...LKHLRLDGNNVTYNSMPPDYVTCLREASDIIFE
LUM2_DANRE	Q501S3	LQYLYLEVNHIQEFNVSSFCRTVGPTS.......YSR...MKILRLDGNKLEYHKLPPDWVFCLRVLHNIYI
LUM2_GASAC	ENSGACESTG00000020601	LQYLYLEANEIQDFNVNSFCRHWGPLS.......YSR...MKILRLEGNKISYQQLPSNWVFCLRVLESIYI
LUM2_ORYLA	ENSORLG00000015717	LQHLYLEANQIQEFNLTSFCRETGPLS.......YSR...LKFLRLDGNKLTYNQLPQDWVYCLRVIQSIYI
LUM2_FUGRU	ENSTRUG00000004773	LQYLYLEANEIKGFNTTSFCREVGPLS.......YSR...MKVLRLDGNNMSYQQIPPDWMLCLRVLQSIYL
FMOD_HUMAN	Q06828	LENLYLQGNRINEFSISSFCTVVDVVN.......FSQ...LQVVRLDGNEMKRSAMPAEAPLCLRLASLIEI
FMOD_PANTR	XP_514114	LENLYLQGNRINEFSISSFCTVVDVVN.......FSK...LQVLRLDGNEIKRSAMPADAPLCLRLASLIEI
FMOD_PONPY	ENSPPYG00000000332	LENLYLQGNRINEFSISSFCTVVDVVN.......FSK...LQVLRLDGNEIKRSAMPADAPLCLRLASLIEI
FMOD_MACMU	ENSMMUG00000021642	LENLYLQGNRINEFSISSFCTVVDVVN.......FSK...LQVLRLDGNEIKRSAMPADAPLCLRLASLIEI
FMOD_BOVIN	P13605	LENLYLQGNRINEFSISSFCTVVDVMN.......FSK...LQVLRLDGNEIKRSAMPADAPLCLRLASLIEI
FMOD_HORSE	A2Q126	LENLYLQGNRINEFSISSFCTVVDVMN.......FSK...LQVLRLDGNEIKRSAMPADAPLCLRLASLIEI
FMOD_CANFA	XP_545677	LENLYLQGNRINEFSISSFCTVVDVMN.......FSK...LQVLRLDGNEIKRSAMPVDAPLCLRLASLIEI
FMOD_MOUSE	P50608	LENLYLQGNRINEFSISSFCTVVDVMN.......FSK...LQVLRLDGNEIKRSAMPVDAPLCLRLANLIEI
FMOD_RAT	P50609	LENLYLQGNRINEFSISSFCTVVDVMN.......FSK...LQVLRLDGNEIKRSAMPVDAPLCLRLASLIEI
FMOD_RABIT	ENSOCUG00000009702	XXXXXXXXXXXXXFSISSFCSVVDVMN.......FSK...LQVLRLDGNQLKRSAMPADAPLCLRLASLIEI
FMOD_MONDO	XP_001364906	LENLYLQGNRINEFSISSFCTVVDVMN.......FSK...LQVLRLDGNEIQRSAVPVDAPLCLRLASLIEI
FMOD_XENTR	ENSXETG00000011835	LQHLYLQGNKIEEFSVKSFCTFVDITS.......FSQ...LQVLRLEGNEIKANAIPPDTPLCLRKADIIHM
FMOD_ANOCA	GENSCAN00000110086	LENLYLQGNRINEFTINSFCTIVDIMN.......FSK...LQVLRLDGNDIHRNNVPSDAPLCLRWASVIEI
FMOD_CHICK	P51887	LENLYLQGNQINEFSISSFCTVVDVMN.......YSR...LQVLRLDGNEIKRNAMPPDAPLCLRRATVIEI
FMOD_DANRE	Q4V9E0	LQHLYLQANQIKEFTLGSFCSVVDVMN.......FSR...LRVLRLEGNEIGAGDVPSEAALCLRLASDIAV
FMOD_GASAC	ENSGACG00000000399	LQHLYLQANQIKEFTLGSFCSAVDVTN.......FSE...LLTLRLDGNQIGPQDIPTDSSLCLRLTSSIEV
FMOD_ORYLA	ENSORLG00000015703	LQHLYLQANQIKEFTLGSFCSIVDVVN.......FSK...LQTLRLDGNEISLQDIPVETAVCLRQAFSIQI
FMOD_FUGRU	ENSTRUG00000005359	LQHLYLQANRIKEFTLGSFCVFTDVTN.......FSR...LQTLRLDGNEIGMEDIPADSSLCLRSASAIDV
FMOD_TETNG	Q4SR97	LQHLYLQANQIKEFTLGSFCTFTDVSS.......FSR...LQTLRLDGNELSQEDIPSDSSLCLRLASAIDV
FMOD2_DANRE	XP_001338027	LENLYLQANKIKEFSVSSFCRVVDTNN.......YSN...LRVLRLEANEITPRDIPNEAVLCLRLATNIDL
FMOD2_GASAC	ENSGACG00000002840	LENLYLHANKIKEFSLSSFCGPISMTN.......FSR...LRMLRLESNEISAKDIPAEAAYCLRRVAFIDV

FMOD2_ORYLA	GENSCAN00000038712	LENLYLHANKIKEFTLSSFCDTVDMTN.......FSR...LKVLRLDANEINARDIPAEAAYCLRRVAFIDV
FMOD2_FUGRU	ENSTRUG00000012573	LENLYLQANRIKEFSLSSFCSTVDMTD.......FSK...MRMLRLDGNQLSAKDIPSEAAYCLRHVASVDV
FMOD2_TETNG	Q4RJX0	LENLYLQANRIKDFSLSSFCRPIDMTN.......FSR...MRMLRLDGNKINAEDIPSEAAYCLRHIVAVDV
KERA_HUMAN	O60938	LQHLHLDHNKIKSVNVSVICPSPSMLPAERD..SFSYGPHLRYLRLDGNEIK.PPIPMALMTCFRLLQAVII
KERA_PANTR	XP_001135135	LQHLHLDHNKIKSVNVSVICPSPSMLPAERD..SFSYGPHLRYLRLDGNEIK.PPIPMALMTCFRLLQAVII
KERA_PONPY	ENSPPYG00000004824	LQHLHLDHNKIKSVNVSVICPSPSMLPAERD..SFSYGPHLRYLRLDGNEIK.PPIPMALMTCFRLLQAVII
KERA_MACMU	ENSMMUG00000012507	LQHLHLDHNKIKSVNVSVICPSPSTLPAERD..SFSYGPHLRYLRLDGNEIK.PPIPMALMTCFRLLQAVII
KERA_BOVIN	O62702	LQHLHLDHNKIRNVNVSVICPSTPTTLPVED..SFSYGPHLRYLRLDGNEIK.PPIPMDLMTCFRLLQAVII
KERA_HORSE	XP_001492790	LQHLHLDHNKIKNVNVSVICPTTTTLPAEQD..IFSYGPHLRYLRLDGNEIK.PPIPMDLMTCFRLLQAVVI
KERA_CANFA	XP_539715	LQHLHLDHNKIKNVNVSVICPPIL..PAEQD..SFGYGPHLRYLRLDGNEIK.PPIPMDLMNCFRLLQAVII
KERA_MOUSE	O35367	LQHLHLDHNKIKNVNMSVICPTTL..RAEQD..AFIHGPQLSYLRLDGNEIK.PPIPIDLVACFKLLQAFII
KERA_RAT	NP_001101557	LQHLHLDHNKIKNVNMSVICPSTL..RAGQD..AFLHGPQLSYLRLDGNEIK.PPIPMDLVACFKLLQAFII
KERA_RABIT	ENSOCUG00000013394	LQHLHLDHNKIKSVNVSLICPTTSTLHTEQD..SFIHGPQLSYLRLDGNEIK.PPIPMDLMTCFRLLQAVII
KERA_DASNO	ENSDNOG00000014143	XXXXXXXXXXXXVNVSVICPTPKMLPGEQD..SFIYGPHLSYLRLDGNDIK.PPIAMDLMTCFRLLQAVII
KERA_MONDO	XP_001363076	LEHLHLDHNRIRSVNSSLMCPPFHAMAGMHD..SFSDGPRLRYLRLDGNEIK.PPIPMDLMVCFRLLQYVVI
KERA_ORNAN	XP_001510956	LEHLHLDHNKIKSVNGSIMCPASVAVSGEHD..SFGYAPRLRYLRLDGNEIK.PPIPMDLMICFRLLQAIVI
KERA_XENTR	ENSXETG00000025479	LERLHLDHNKIKNINGNILCPEVVKEEFDPH...FPHGPRLRYLRLDGNEVQ.PPIPLELMICFRLLQTVVI
KERA_ANOCA	GENSCAN00000094131	LEHLHLDNNKISSVNGTQVCPIPIPGDYVNE....RNLPRLRYLRLDGNEIK.PPIPFDLMLCFRLLQAVVI
KERA_CHICK	O42235	LEHLHLDHNRIKSVNGTQICPVSIAVAEDYG..LYGNIPRLRYLRLDGNEIQ.PPIPLDIMICFQLLQAVVI
KERA_COTJA	Q9DE66	LEHLHLDHNRIKSVNGTQICPVSIAVAEDYG..LYGNIPRLRYLRLDGNEIQ.PPIPLDIMICFQLLQAVVI
KERA_DANRE	Q5RI43	LEHLHLDHNKIKSVNSSDICPPGVLDDHLEE.....KSPRLRYLRLDGNEIQ.PPIPRELMTCFRLLRAVVI
KERA_GASAC	ENSGACG00000019816	LEQLHLDHNNIKTVSGANICPISFEAMQDSD...NDSVPRLRYLRLDGNTIK.PPIPRDVILCFRLLRSIVI
KERA_ORYLA	ENSORLG00000015326	LEHLHLDHNKITYVNGSKICPVSVDAVDDSL...NESVPQLRYLRLDGNEIN.PPIPRDVITCFRFLRSIVI
KERA_FUGRU	ENSTRUG00000013697	LEHLHLDHNNIKSVDGSNVCPVSIDSMDDSV...NDNIPRLRYLRLDGNEIK.PPIPREVILCFRLLRSIVI
KERA_TETNG	Q4SBU9	LEQLHLDHNNIKSIDGSNVCPVSTDTTDDFV...SSNVPRLRYLRLDGNDIK.PPIPREVILCFRLLRSIVI
PRELP_HUMAN	P51888	LEHLYLNNNSIEKINGTQICPNDLVAFHDFS.SDLENVPHLRYLRLDGNYLK.PPIPLDLMMCFRLLQSVVI
PRELP_PANTR	XP_525032	LEHLYLNNNSIEKINGTQICPNDLVAFHDFS.SDLENVPHLRYLRLDGNYLK.PPIPLDLMMCFRLLQSVVI
PRELP_PONPY	ENSPPYG00000000331	LEHLYLNNNSIEKINGTQICPNDLVAFHDFS.SDLENVPHLRYLRLDGNYLK.PPIPLDLMMCFRLLQSVVI
PRELP_MACMU	ENSMMUG00000021641	LEHLYLNNNSIEKINGTQICPNNLVAFHDFS.SDLENVPHLRYLRLDGNYLK.PPIPLDLMMCFRLLQSVVI
PRELP_BOVIN	Q9GKN8	LEHLYLNNNSIEKINGTQICPNNIVAFHDFS.SDLEHVPHLRYLRLDGNYLK.PPIPLDLMMCFRLLQSVVI
PRELP_HORSE	ENSECAG00000018126	LEHLYLNNNSIEKINGTQICPNNLVAFHDFS.SDLENVPHLRYLRLDGNYLK.PPIPLDLMMCFRLLQSVVI
PRELP_CANFA	XP_545678	LEHLYLNNNSIEKINGTQICPNNLVAFHDFS.SDLENVPHLRYLRLDGNHLK.PPIPLDLMMCFRLLQSVVI
PRELP_MOUSE	Q9JK53	LEHLYLNNNSIEKINGTQICPNNLVAFHDFS.SDLENVPHLRYLRLDGNFLK.PPIPLDLMMCFRLLQSVVI
PRELP_RAT	Q9EQP5	LEHLYLNNNSIEKINGTQICPSNLVAFHDFS.SDLENVPHLRYLRLDGNFLK.PPIPLDLMMCFRLLQSVVI
PRELP_MONDO	XP_001370119	LEHLYLNNNEIQKINGTQICPNNLVAFHDFS.SDLENVPHLRYLRLDGNQLK.PPIPLDLMMCFRLLQSVII
PRELP_ORNAN	XP_001518615	LEHLYLNGNEINKINGTQICPNNLVTFHDFS.SDLENVPHLRYLRLDGNLLK.PPIPIDLMLCFRLLQSVVI
PRELP_XENTR	A4IIL0	LEHLYLNDNIIEKINGTEICPAPLVPFDFSS.RDLSSVPRLRYLRLDGNQLK.PPIPLDVIMCFRLLQSVVI
PRELP_ANOCA	GENSCAN00000110083	LEHLYLNDNSIEKINGTQICPISLMTFQEPS.SDLQNVPRLRYLRLDGNHLK.PPIPLDLMMCFRLLQSVVF

PRELP_CHICK	XP_418054	LEHLYLNNNSIEKINGTQICPTSLMSIQDFSPSDLDSVPRLRYLRLDGNLLK.PPIPLDLMMCFRLLQSVVF
PRELP_DANRE	A8BBF7	LEQLHLNNNNIESVNGTEICPPHNVH.......DENGAPKLRYLRLDGNHLS.PPVPLDVIMCFRHLHAIVI
PRELP_GASAC	ENSGACG00000000397	LEHLHLNHNSIESINGTQICPYSLQAD....PSDLSLVPSLRYLRLDGNQLS.PPIPLDVIMCFKQLHSIVI
PRELP_FUGRU	ENSTRUG00000004773	LEHLHLNHNQIESINGTQICPVTLEDEL....ADESLVPRLRYLRLDGNHLS.PPIPMSVIMCFRHLRSIVL
PRELP_TETNG	GSTENG00014012001	LEHLHLNHNHIESINGTQICPVTLEDEL....QDESLVPRLRYLRLDGNNLN.PPIPMDVIMCFRHLRSIVL
OMD_HUMAN	Q99983	LEHLYLQNNEIEKMNLTVMCPSIDPLH.......YHH...LTYIRVDQNKLK.EPISSYIFFCFPHIHTIYYGEQRS...
OMD_PANTR	XP_001146269	LEHLYLQNNEIEKMNLTVMCPSIDPLH.......YHH...LTYIRVDQNKLK.EPISSYIFFCFPHIHTIYYGEQRS...
OMD_PONPY	ENSPPYG00000019378	LEHLYLQNNEIEKMNLTVMCPSIDPLH.......YHH...LTYIRVDQNKLK.EPISSYIFFCFPHIHTIYYGEQRS...
OMD_MACMU	XP_001103423	LEHLYLQNNDIEKMNLTVMCPSIDPLH.......HHH...LTYIRVDQNKLK.EPISSYIFFCFPHIHTIYYGEQRS...
OMD_BOVIN	O77742	LEHLYLENNEIENVNVTVMCPSVDPLH.......YHH...LTHIRIDQNKLK.APISSYIFLCFPHIHTIYYGEQQS...
OMD_HORSE	ENSECAG00000014287	LEHLYLENNEIENINLTVMCPSVDPLH.......YHH...LTYIRVDQNKLK.EPINSYIFLCFPHIHSIYYGEQRS...
OMD_CANFA	XP_853328	LQHLYLQNNEIESINVTVMCPSVDPLH.......YHH...LTYIRLDQNKLK.EPITSYISLCFPYIHTIYYGEQRS...
OMD_MOUSE	O35103	LEHLYLQNNEIESINVTMICPSPDPVH.......HHH...LTYLRVDQNKLK.EPISSYIFFCFPRIHSIYYGEQRS...
OMD_RAT	Q9Z1S7	LEHLYLQNNEIQSINVTMMCPSLDPLH.......HHH...LTYLRVDQNKLK.EPISSYIFFCFPRIHSIYYGEQRS...
OMD_DASNO	ENSDNOG00000010606	LEHLYIENNEIEDINITVICPSIDPLY.......YHH...LTYIRVDQNKLK.EPINSYVFACFPHIHAVYYGEQRS...
OMD_ORNAN	XP_001512765	LEHLYIEDNEIENVNITLLCPSIDPLH.......YHH...LTYIRLEKNRLK.APISTYVFFCFPHMHTIYYGEQKN...
OMD_XENTR	GENSCAN00000046434	LQHLYIQDNELESLNMTIMCPKIDHFN.......TNH...LTYLRVDQNKLQ.APISTLAYLCFPNMQRIYYGEQKY...
OMD_ANOCA	GENSCAN00000066144	LQHLYIEDNEIEVMNITLMCPSLDPLN.......FSH...LTYIRVDQNKLT.APLSTYAFFCFPHIRSIYYGEQKL...
OMD_CHICK	GENSCAN00000036882	LQHLYIEDNDIEMINVTLMCPSIDLMN.......INQ...LTYIRVDQNKLT.TPISTYAFFCFPHIRTIYYGEQNS...
OMD_DANRE	Q1LV49_DANRE	LEHLYLNHNDFKDLNISLMCPSLDLGH.......PNM...LTYIRLDNNKLS.GPVDYYAYRCFPRLIMIFYGHQRK...
KERAL_PETMA	GENSCAN00000147526	LVQLHLDENDIEDVNTTALCRPEGRES........SR...LSYFRLDKNPIM.ESPQAPLMHCFPYLQPMF

Class III: OGN and OGN2 (mimecans/osteoglycins); EPYC (epiphycan); OPTC (opticin); EPYL (epiphycan-like sequence from lamprey)

OGN_HUMAN	P20774	LRVIHLQFNNIASITDDTFCKANDTSYI......RDR...IEEIRLEGNPIVLGKHPNS.FICLKRLPIGSYF
OGN_PANTR	XP_001145422	LRVIHLQFNNIASITDDTFCKANDTSYI......RDR...IEEIRLEGNPIVLGKHPNS.FICLKRLPIGSYF
OGN_PONPY	Q5RBL2	LRVIHLQFNNIASITDDTFCKANDTSYI......RDR...IEEIRLEGNPIVLGKHPNS.FICLKRLPIGSYF
OGN_MACMU	ENSMMUG00000004634	LRVIHLQFNNIASITDDTFCKANDTSYI......RDR...IEEIRLEGNPVVLGKHPNS.FICLKRLPIGSYF
OGN_BOVIN	P19879	LRVIHLQFNNITSITDDTFCKANDTSYI......RDR...IEEIRLEGNPVILGKHPNS.FICLKRLPIGSYI
OGN_HORSE	ENSECAG00000017802	LRVIHLQFNNITSITDDTFCKANDTRYI......RVR...IEEIRLEGNPIVLGKHPNS.FICLKRLPVGSYV
OGN_CAPHI	A1YZ35	LRVIHLQFNNITSITDDTFCKANDTSYI......RDR...IEEIRLEGNPIILGKHPNS.FICLKRLPVGSYI
OGN_CANFA	XP_853340	LRVIHLQFNNITSITDDTFCKANDTRYI......RDR...IEEIRLEGNPIVLGKHPNS.FICLKRLPVGSYF
OGN_MOUSE	Q62000	LRVIHLQFNSISSLTDDTFCKANDTRYI......RER...IEEIRLEGNPIALGKHPNS.FICLKRLPIGSYF
OGN_RAT	NP_001099573	LRVIHLQFNSISSITDDTFCKANDTRYI......RER...MEEIRLEGNPIALGKHPNS.FICLKRLPTGSYF
OGN_RABIT	Q8MJF1	LRVIHLQFNNITSITDDTFCKANDTRYI......RDR...IEEIRLEGNPIALGKHPNS.FICLKRLPIGTYF

OGN_ORNAN	XP_001512743	LRVIHLQYNNITSITDDTFCKSNDTRYI......RNR...VDEIRMEGNPIILGKHPNS.FICLKKLPIGTYY
OGN_XENLA	Q7ZYH2	LKILHLQQNNITSITDDTFCKSNDTRYI......RSH...MAEIRMEGNPVILGKHPNS.FTCLKTLPSGSYFK
OGN_XENTR	ENSXETG00000027245	LRILHLQQNGISSITDDTFCKSNDTHYV......RSH...MDEIRMEGNPVILGKYPNS.FTCLKTLPSGSYFK
OGN_ANOCA	GENSCAN00000066153	LRILHLQYNNITTITAETFCKGNNQTRYV.....RQR...MDEIRMEGNPVILGKYPNA.FMCLRMLPIGTYF
OGN_CHICK	Q9W6H0	LRILHLQHNNITTINDDTFCKSNNTRYI......RTR...MDEIRMEGNPILLAKHVNA.FSCLRTLPVGTYY
OGN_COTJA	Q9DE65	LRILHLQHNNITTITDDTFCKSNNTRYI......RTR...MDEIRMEGNPILLAKHVNA.FSCLKTLPVGTYY
OGN_DANRE	Q1LV51	VRTLHLQNNNISTVSTDTFCKSNDTYYI......RPN...MNEIRMDGNPINLGQYPNS.FICLQSLPIGRYQ
OGN_GASAC	ENSGACG00000004073	VRILHLQNNNITEVNVDTFCRSNDTYYL......RPS...LSEVRMDGNPVVLSKYPDS.FTCMKVLPIGQYR
OGN_ORYLA	ENSORLG00000019880	VRILHLQNNNITNVTIDTFCKSDNNYYL......RPS...LSEVRLDGNPVVLSKYPDS.FTCMKVLPVGRYR
OGN_FUGRU	ENSTRUG00000009433	VRIVHLQNNNITEVNVNTFCKSNDTYYL......RPS...LSEVRLDGNPVMLSKYHDS.FTCMNVLPVGKYR
OGN_TETNG	Q4TBJ6	VRILHLQNNNITEVNKDTFCKSNNTYYL......RPS...LSEVRLDGNPAVLSTYADS.FTCMKVLPVGKYR
OGN2_DANRE	XP_684029	LRVVHLHNNNITSLTDDTFCKGNSTHYI......RHN...MQEVRLDGNPITLAQHPNS.FICLRALPIGHYK
OGN2_FUGRU	ENSTRUG00000011016	LHVVHLQNNKIEAITDETFCKGNTSYYV......RTK...MEEVRLDGNPVVLANYPYS.FICLQSLPVGWYN
OGN2_TETNG	Q4T4G9	LHIVHLNNNKIAAITDETFCKGNTSYYV......RTK...MDEVRLDGNPVVLANYPYS.FICLKSLPVGWYN
EPYC_HUMAN	Q99645	LRALHLQNNNIMEMHEDTFCNVKNLTYI......RKA...LEDIRLDGNPINLSKTPQA.YMCLPRLPIGSLV
EPYC_PANTR	XP_001134960	LQALHLQNNNILEMHEDTFCNVKNLTYI......RKA...LEDIRLDGNPINLSKTPQA.YMCLPRLPVGSLV
EPYC_PONPY	ENSPPYG00000004823	LRALHLQNNNILEMHEDTFCNVKNLTYI......RKA...LEDIRLDGNPINLSKTPQA.YMCLPRLPVGSLV
EPYC_MACMU	ENSMMUG00000011300	LRALHLQNNNILEMHEDTFCNVKNLTYI......RKA...LEDIRLDGNPINLSKTPQA.YMCLPRLPVGSLV
EPYC_BOVIN	P79119	LRALHLQNNNILEMHEDTFCNVKNLTYI......RKA...LEDIRLDGNPINLSKTPQA.YMCLPRLPVGSLV
EPYC_HORSE	XP_001492764	LRALHLQNNNILEMHEDTFCNVKNLTYI......RKA...LEDIRLDGNPINLSKTPQA.YLCLPRLPIGSLV
EPYC_CANFA	XP_539714	LRALHLQNNNILEMHEDTFCNVKNLTYI......RKA...LEDIRLDGNPINLSKTPQA.YMCLPRLPIGSLV
EPYC_MOUSE	P70186	LRALHLQNNDILEMHEDTFCNVKNLTYV......RKA...LEDIRLDGNPINLSRTPQA.YMCLPRLPIGSFI
EPYC_RAT	NP_001101558	LQALHLQNNDILEMHEDTFCNVKNLTYV......RKA...LEDIRLDGNPINLSKTPQA.YMCLPRLPIGSFI
EPYC_MONDO	ENSMODG00000009682	LRALHLQNNNILEMHEDTFCNVKNLTYV......RKA...LEDIRLDGNPINLSKTPQA.YMCLPRLPIGSLV
EPYC_ORNAN	XP_001512092	LQALHLQNNNILEMHEDTFCNVKNLTYV......RKA...LEDIRLDGNPINLSKTPQA.YMCLPRLPVGSLI
EPYC_XENLA	Q66KS3	LQSLHLQDNNIQEMHEDTFCKMKDWTYA......RRT...LEDIRLDGNPINLSRTPQA.FMCLPRIPIGRLV
EPYC_XENTR	Q6DK81	LQSLHLQDNNIQEMHEDTFCKMKDWTYA......RRA...LEDIRLDGNPINLSRTPQA.FMCLPRIPIGRLV
EPYC_ANOCA	GENSCAN00000094132	LQSLHLQNNNIQEMHEDTFCKMKDYSYI......RKS...LEDIRLDGNPINLSKTPYA.YMCLPRLPVGSLF
EPYC_CHICK	Q90944	LQALHLQNNNIQEMHEDTFCKMRDFSYV......RRA...LEDIRLDGNPINLSKTPYA.YMCLPRLPVGNLI
EPYC_DANRE	Q568A7	LRSLHLQNNNIQMIHEDTFCNPHDLNYI......RNA...LEDVRLDGNPINLSRTPQA.YICLPRIPIGNLF
EPYC_GASAC	ENSGACG00000019817	LRSLHLQRNNIQMMHGDTFCNLKDFNYI......RNA...LEDIRLDGNPINLSRTPQA.YVCLPRIPIGNLI
EPYC_ORYLA	ENSORLG00000015331	LRSLHLQRNNIQMMHEDTFCNLNDFNYI......RNA...LEDIRLDGNPINLSKTPQA.YVCLPRIPIGNLI
EPYC_FUGRU	ENSTRUG00000013658	LRSLHLQRNNIQSMHEDTFCNLKDFNYI......RNA...LEDIRLDGNPINLSRTPQA.YICLPRIPIGDLV
EPYC_TETNG	Q4SBV0	LRSLHLQRNNIQSMHEDTFCNLKDFNYI......RNA...LEDIRLDGNPINLSRTPQA.YICLPRIPIGDLV
OPTC_HUMAN	Q9UBM4	LRSVHLQNNLIETMQRDVFCDPEEHKHT......RRQ...LEDIRLDGNPINLSLFPSA.YFCLPRLPIGRFT
OPTC_PANTR	XP_001155711	LRSVHLQNNLIETMQRDVFCDPEEHKHT......RRQ...LEDIRLDGNPINLSLFPSA.YFCLPRLPIGRFT
OPTC_PONPY	ENSPPYG00000000330	LRSVHLQNNLIETMQRDVFCDPEEHKHT......RRQ...LEDIRLDGNPINLSLFPSA.YFCLPRLPIGRFT

OPTC_MACMU	ENSMMUG00000021640	LRSVHLQNNLIETMQRDVFCDPEEHKYT......RRQ...LEDIRLDGNPINLSLFPSA.YFCLPRLPIGRFV
OPTC_BOVIN	P58874	LRSLHLQNNMIETMQRDAFCDAEEHRHT......RRP...LEDIRLDGNPINLSLFPSA.YFCLPRLPTGRFV
OPTC_HORSE	XP_001488793	LRSLHLQNNRIETMQRDAFCDPEEHKYT......RRR...LEDIRLDGNPINLGLFPSA.YFCLPRLPTGRFA
OPTC_PIG	Q8MIS4	LRSLHLQNNMIETLQRDTFCDPEEHKHT......RRQ...LEDIRLDGNPINLSLFPSA.YFCLPRLPIGRFT
OPTC_CANFA	P83286	LRSLHLQNNLIETMQTDAFCDPEEHKHS......RRW...LEDIRLDGNPINLGLFPSA.YFCLPRLPTGHCC
OPTC_MOUSE	Q920A0	LRSLHLQNNMIETMESDTFCDTGEHRHE......RRQ...LEDIRLDGNPINLSLFPEA.YFCLPRLPVGHFT
OPTC_RAT	ENSRNOG00000003059	LRSLHLQNNKIEAMESDTFCDTTEHRHER.....RRQ...LEDIRLDGNPINLSLFPEA.YFCLPRLPVGRFT
OPTC_MONDO	XP_001370152	LRSLHLQNNHIQKIQEGTFCDAEDHKYV......RRV...LEDIRLDGNPINLSLTPNA.YFCLPRLPIGSYI
OPTC_XENTR	Q28HP1	LRSLHLKNNNIQTMNLETFCESRDPTFI......RRN...LEDIRVDENPINLSKFVNG.YFCLVRLPTGSYY
OPTC_ANOCA	GENSCAN00000110067	LRSLHLQNNHIQTLHEDTFCDGQDHSHI......RRA...LEDIRLDGNPINLSLYPNA.YFCLPRLPTGHF
OPTC_CHICK	Q6YEX8	LRSLHLQNNNIQTMHEDTFCNSQDQSHV......RRA...LEDIRLDGNPINLSLFPNA.YFCLPRLPTGHFL
OPTC_DANRE	Q15JE7	LRVLHLQNNNIQSITQDTFCNSHDKNYI......RKA...LEDIRLDGNPVDINLYPQA.YFCLPRLPVGTPV
EPYL_DANRE	GENSCAN00000105619	LRVLHLQNNNIQQIREDTFCKPKELSYF......RKA...LEDVRLDGNPVNLSDAPEA.YTCLPRIPTGATF
