# Supplementary figures and images for: LRRCE: a leucine-rich repeat cysteine capping motif unique to the chordate lineage
Source: BMC Genomics. 2008 Dec 12;9:599. doi: 10.1186/1471-2164-9-599 (PMC2637281; doi:10.1186/1471-2164-9-599)

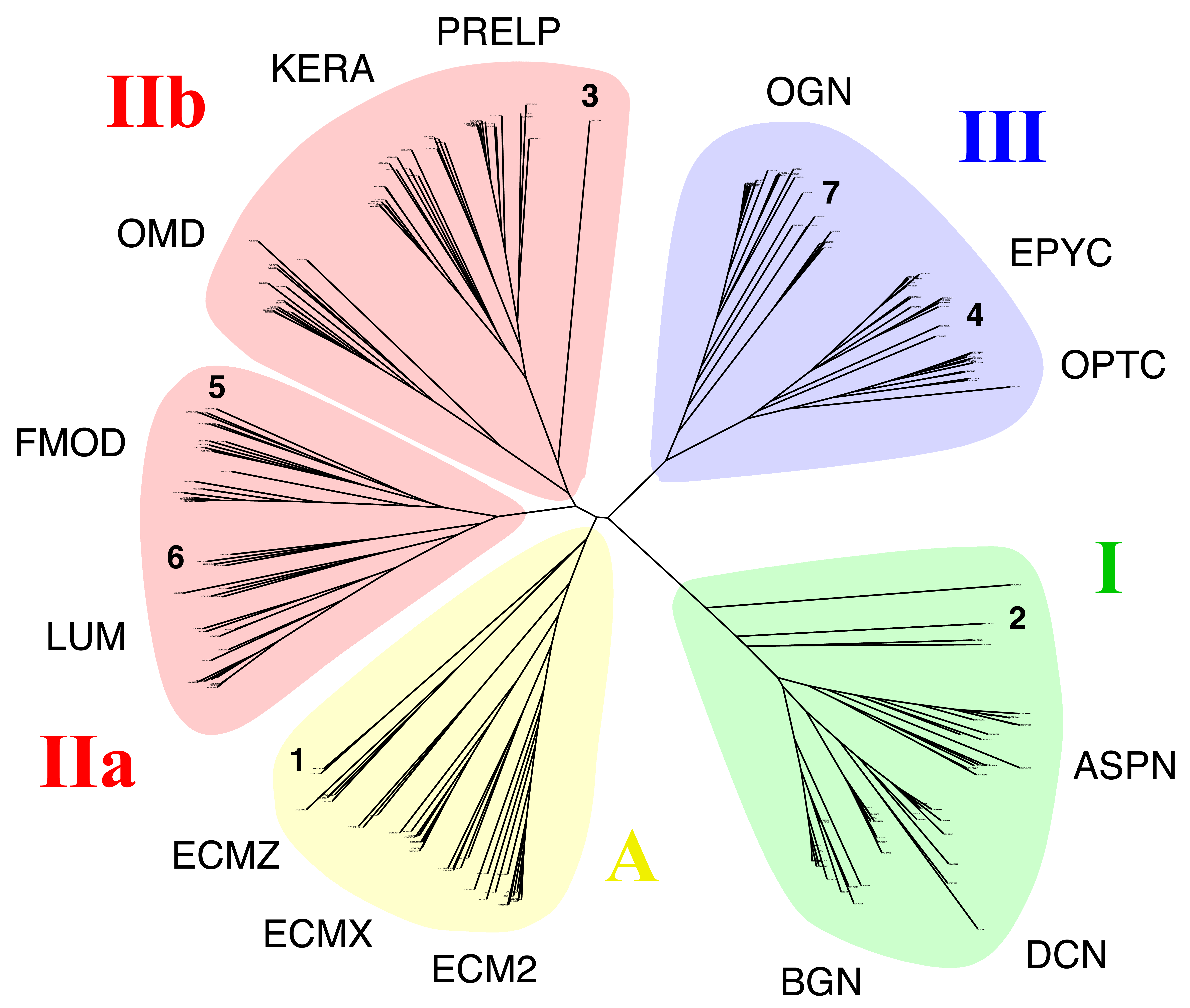

Supplement: Additional file 2 — high-resolution version of Figure 4. Larger version of Figure 4, with legible sequence names at the end of the phylogenetic tree branches. [file 1471-2164-9-599-S2.tiff]
